# Genetic Dissection of Bloom Time in Low Chilling Sweet Cherry (*Prunus avium* L.) Using a Multi-Family QTL Approach

**DOI:** 10.3389/fpls.2019.01647

**Published:** 2020-01-09

**Authors:** Alejandro Calle, Lichun Cai, Amy Iezzoni, Ana Wünsch

**Affiliations:** ^1^ Unidad de Hotofruticultura, Centro de Investigación y Tecnología Agroalimentaria de Aragón (CITA), Zaragoza, Spain; ^2^ Instituto Agroalimentario de Aragón-IA2, (CITA-Universidad de Zaragoza), Zaragoza, Spain; ^3^ Department of Horticulture, Michigan State University, East Lansing, MI, United States

**Keywords:** sweet cherry, bloom time, chilling requirements, SNPs, quantitative trait loci

## Abstract

Bloom time in sweet cherry (*Prunus avium* L.) is a highly heritable trait that varies between genotypes and depends on the environmental conditions. Bud-break occurs after chill and heat requirements of each genotype are fulfilled, and dormancy is released. Bloom time is a critical trait for fruit production as matching cultivar adaptation to the growing area is essential for adequate fruit set. Additionally, low chilling cultivars are of interest to extend sweet cherry production to warmer regions, and for the crop adaptation to increasing winter and spring temperatures. The aim of this work is to investigate the genetic control of this trait by analyzing multiple families derived from the low chilling and extra-early flowering local Spanish cultivar ‘Cristobalina’ and other cultivars with higher chilling requirements and medium to late bloom times. Bloom time evaluation in six related sweet cherry populations confirmed a high heritability of this trait, and skewed distribution toward late flowering, revealing possible dominance of the late bloom alleles. SNP genotyping of the six populations (n = 406) resulted in a consensus map of 1269 SNPs. Quantitative trait loci (QTL) analysis using the Bayesian approach implemented by FlexQTL™ software revealed two major QTLs on linkage groups 1 and 2 (qP-BT1.1m and qP-BT2.1m) that explained 47.6% of the phenotypic variation. The QTL on linkage group 1 was mapped to a 0.26 Mbp region that overlaps with the *DORMANCY ASSOCIATED MADS-BOX (DAM)* genes. This finding is consistent with peach results that indicate that these genes are major determinants of chilling requirement in *Prunus*. Haplotype analysis of the linkage group 1 and 2 QTL regions showed that ‘Cristobalina’ was the only cultivar tested that contributed early bloom time alleles for these two QTLs. This work contributes to knowledge of the genetic control of chilling requirement and bloom date and will enable marker-assisted selection for low chilling in sweet cherry breeding programs.

## Introduction

Bloom time (BT) is an important horticultural trait in temperate fruit tree species like sweet cherry (*Prunus avium* L.). Cultivar adaptation to climatic conditions in the growing area is essential for flower production and fruit set. Early blooming cultivars are susceptible to spring frost damage in cold regions (Luedeling, 2012), while late blooming cultivars can exhibit irregular floral development and low fruit set due to warm temperatures during the flowering period (Mahmood et al., 2000; Atkinson et al., 2013). The biological control of BT is complex and is known to depend on environmental signals during the winter and spring seasons (Abbott et al., 2015; Fadón and Rodrigo, 2018). Fruit trees like sweet cherry require a period of chilling temperatures followed by a period of warm temperatures to induce blooming (Lang et al., 1987). In *Prunus* species, several studies indicate that BT is more dependent on chilling than on heat requirement and that there is large variation in these requirements among individuals of the same species (Campoy et al., 2011; Okie and Blackburn, 2011; Sánchez-Pérez et al., 2012; Castède et al., 2014).

Several genetic studies in *Prunus* have focused on understanding the genetic control of chilling (CR) and heat requirements (HR) contributing to the differences in BT (reviewed in Abbott et al., 2015). BT in *Prunus* is a quantitative trait with high heritability (Anderson and Seeley, 1993; Dirlewanger et al., 2012; Castède et al., 2014), and genetic approaches have led to the identification of quantitative trait loci (QTLs) associated with this trait. BT QTLs have been identified on all *Prunus* linkage groups (LGs) (reviewed in Salazar et al., 2014; Abbott et al., 2015), but major QTLs have been found on LG1 (Fan et al., 2010; Zhebentyayeva et al., 2014; Bielenberg et al., 2015) and LG4 (Sánchez-Pérez et al., 2012; Dirlewanger et al., 2012; Castède et al., 2014) in all the *Prunus* crop species evaluated to date. In sweet and sour (*Prunus cerasus*) cherries, several QTLs have been identified for BT and CR. In sweet cherry, Dirlewanger et al. (2012) mapped two major BT QTLs on LGs 1 and 4 and three minor QTLs on LGs 5, 6 and 7. Castède et al. (2014) using three to six years data and two F_1_ populations identified BT and CR QTLs in all LGs, with a major and stable QTL for both traits overlapping on LG4. Castède et al. (2014) also detected minor QTLs for both CR and BT on LGs 1 and 7, highlighting the influence of CR in BT in this species. In sour cherry, Wang et al. (2000) investigated BT QTL using an F_1_ population and 3-year data, and two major QTLs were identified on LGs 1 and 2. Another QTL study in sour cherry revealed four QTLs for BT on LGs 1, 2, 4, and 5; the most significant allele for LG4 QTL was associated with almost three days bloom delay (Cai et al., 2018).

Candidate genes have been reported for the *Prunus* BT and CR QTL that maps to LG1 (Yamane et al., 2011; Zhebentyayeva et al., 2014; Castède et al., 2015). In peach, a tandem set of six *DORMANCY ASSOCIATED MADS-BOX (DAM)* genes have been identified in this region (Zhebentyayeva et al., 2014; Romeu et al., 2014; Bielenberg et al., 2015). Studies of these genes was facilitated by the study of a peach non-dormancy mutant termed *evergrowing* peach mutant (*evg*) that has a deletion within this QTL region (Bielenberg et al., 2008). Expression analyses in peach reported that *DAM5* and *DAM6* are not expressed under chilling temperatures (Jimenez et al., 2010) whereas the expression of *DAM4* and *DAM6* are activated by short photoperiods (Li et al., 2009) suggesting that *DAM5* and *DAM6* are the main genes underlying this *Prunus* LG1 CR QTL (Yamane et al., 2011). For the major *Prunus* BT QTL located on LG4, genes related to temperature sensing (*ARP4, EMF2, NUA*, and *PIE1*) and the gibberellin pathway (*GA2ox* and *KS* genes) have been proposed as the most promising candidates to control BT (Dirlewanger et al., 2012; Castède et al., 2015).

Most BT QTL studies in *Prunus* have been done using a single bi-parental population. This strategy is limited because only alleles present and segregating in the two parental cultivars can be detected (Bink et al., 2014). However, knowledge of the effects of these alleles in different genetic backgrounds and other loci not segregating in the bi-parental cross are needed to fully implement marker-assisted selection (MAS). The development of QTL mapping approaches based on multi-parental populations allow the identification a larger number of QTLs and QTL alleles improving the application of these results in MAS for a larger number of genetic backgrounds (Pauly et al., 2012). The Bayesian QTL mapping approach implemented by FlexQTL^™^ (Bink et al., 2008 and Bink et al., 2014) allows analyzing multiple pedigree-linked progenies at the same time; reducing the limitations derived from QTL analyses using single populations. This approach has been successfully used in recent years for QTL analyses of different traits in *Rosaceae* species, such as sweet cherry (Rosyara et al., 2013), apple (Bink et al., 2014; Guan et al., 2015; Allard et al., 2016; Di Guardo et al., 2017; Howard et al., 2018), peach (Fresnedo-Ramírez et al., 2015; Fresnedo-Ramírez et al., 2016; Hernández Mora et al., 2017), and strawberry (Roach et al., 2016; Mangandi et al., 2017; Anciro et al., 2018).

Furthermore, previous QTL analyses in sweet cherry used cross-pollinated F_1_ populations. Self-pollination is often not possible in sweet cherry due to the gametophytic self-incompatibility system operating in this species (Herrero et al., 2017). However, natural self-compatible sweet cherry mutants like ‘Cristobalina’ (Wünsch and Hormaza, 2004) or other self-compatible sweet cherry accessions can be used to generate F_2_ populations which can be used for genetic mapping studies. The self-compatible local cultivar ‘Cristobalina’ and its self-compatible descendant, the selection ‘BC-8’, were used to develop two self-pollinated populations for genetic analysis. Genetic maps of these sweet cherry populations were constructed, and their level of homozygosity was previously reported (Calle et al., 2018). These were the first F_2_ populations used for genetic map construction in this species and are now available for QTL analysis. The Spanish cultivar ‘Cristobalina’ comes from the Mediterranean area, and in addition to being self-compatible (Wünsch and Hormaza, 2004; Cachi and Wünsch, 2011; Ono et al., 2018), it has a low CRs (< 550 h; Tabuenca, 1983; Alburquerque et al., 2008) and extra early flowering and maturity dates. These characteristics make ‘Cristobalina’ an interesting breeding cultivar. Cultivars with low CRs often show early flowering (Alburquerque et al., 2008) and this low chilling requirement is of high interest for extending cherry growing to regions with warmer winters, and in the current context of global warming as a source of adaptation to low chilling.

The objective of this work was to investigate the genetic basis of BT in different genetic backgrounds, including in the low chilling ‘Cristobalina’. To achieve this objective, six related sweet cherry populations that descend from ‘Cristobalina’ and other sweet cherry cultivars with mid and late BTs were used. Four years of BT data from these families was used for QTL analysis using a Bayesian approach implemented in FlexQTL™. Two self-pollination populations were also used to investigate the genetic effects of the QTL alleles. The results obtained broaden the understanding of the genetic control of CR and BT in this species and will allow the implementation for MAS of these traits from this and related plant material.

## Materials and Methods

### Plant Materials

**Figure 1:**
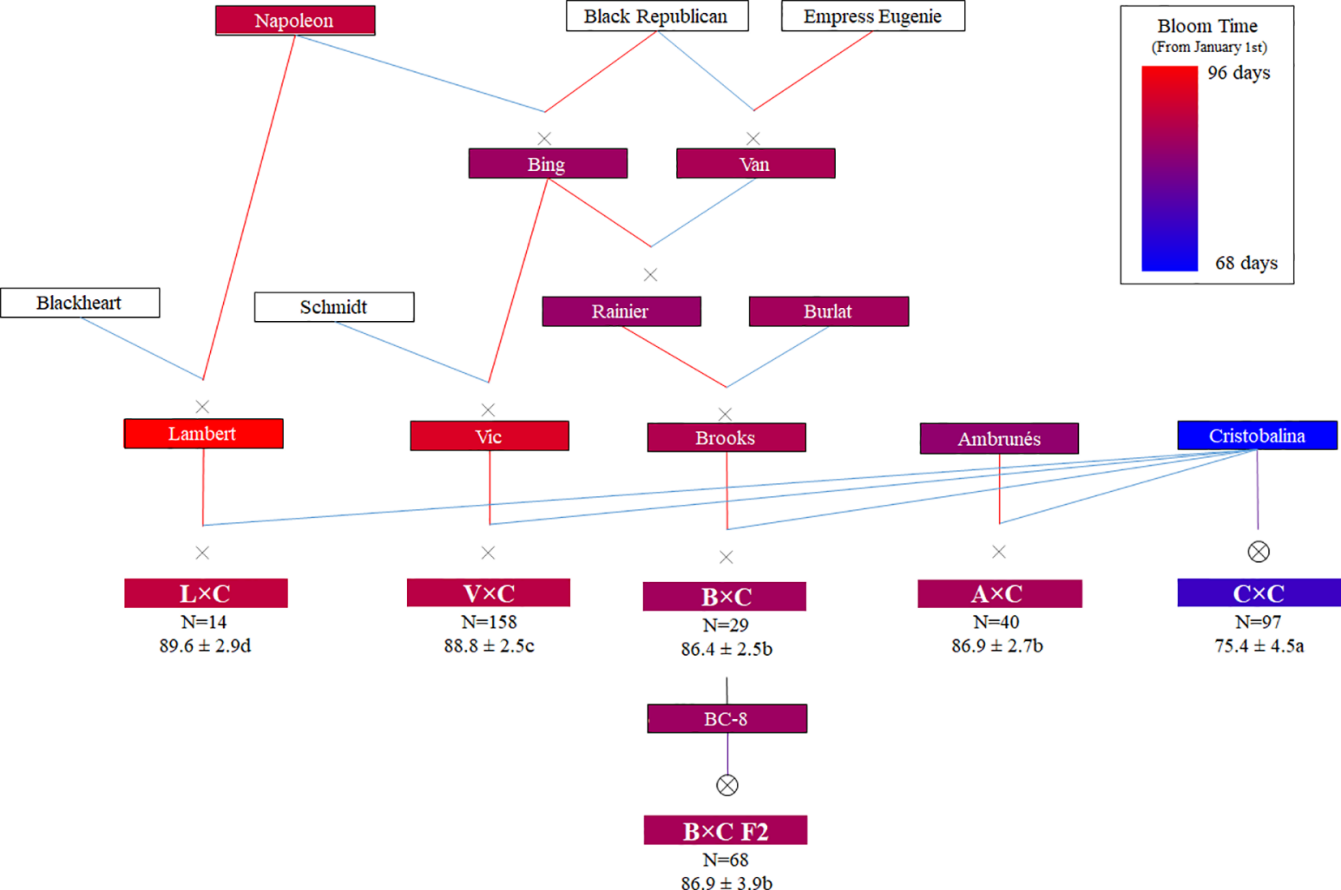
Families, parental and ancestor cultivars used in this work with their known pedigrees (Red and blue lines indicate female and male parent, respectively). Color code (from Pedimap software; Voorrips, 2007) indicates the BLUP (Best linear unbiased prediction) of bloom time values expressed in Julian days (JDs). In the families, the number of individuals (N), mean value of bloom in JDs and significant differences of bloom time among different families (ANOVA and Tukey Test; P < 0.05) are also shown.

Six related sweet cherry full-sib families (N = 406), along with six parental cultivars and five ancestor cultivars, were used in this study (**Figure 1**). Four of these families come from cross-pollinations (F_1_), namely ‘Vic’ × ‘Cristobalina’ (V×C; N = 158), ‘Ambrunés’ × ‘Cristobalina’ (A×C; N = 40), ‘Brooks’ × ‘Cristobalina’ (B×C; N = 29) and ‘Lambert’ × ‘Cristobalina’ (L×C; N = 14). The remaining two populations (F_2_) come from the self-pollination of ‘Cristobalina’ (C×C; N = 97) and ‘B-C8’ (B×C F2; N = 68; named B×C2 in Calle et al., 2018), which is a selection from the progeny of ‘Brooks’ **×** ‘Cristobalina’ (**Figure 1**). All these trees derive from controlled cross- and self-pollinations made from 2008 to 2010 and are located at the experimental orchards of CITA de Aragón in Zaragoza (Spain).

### Bloom Time Phenotyping

BT was evaluated for each progeny and the parental cultivars during 4 years (2015 to 2018). During the flowering season, blooming stage was evaluated 3 days per week (every 2–3 days) and BT was recorded when approximately 75% of the floral buds reached stage F (full bloom; Baggiolini, 1980). BT data were expressed as Julian Days (JD; days from January 1^st^). The best linear unbiased prediction (BLUP) value among years was calculated using the *lme4* package of R 3.4.1 software (Bates et al., 2015; R Core Team, 2017).

Mean, minimum and maximum values and the distribution of BTs were estimated in each population per year and for the BLUP values. Mean BTs were then compared between families using analysis of variance (ANOVA) and Tukey post-hoc test (p < 0.05) for BLUP values. *Pearson* correlation coefficient of BTs between years and BLUP values were also estimated. Statistical analyses were carried out using SPSS statistics v21.0.0 software (IBM, Chicago, IL, USA).

Broad-sense heritability (*H^2^*) was calculated using the equation: *H^2^* = $\frac{\sigma_{g}^{2}}{\sigma_{g}^{2}{+}_{n}^{\sigma_{e}^{2}}}$, where $\sigma_{g}^{2}$ is the variance of genotype effect, $\sigma_{e}^{2}$is the variance of the residual term, and *n* is the number of years. *H^2^* were calculated using R v3.4.1 (R Core Team, 2017).

### Genotyping and QTL Analysis

Genotypes of 417 individuals that include the six populations described above, their parental and ancestor cultivars (**Figure 1**) were used for QTL analysis. All these plant material had been previously genotyped using the RosBREED Illumina cherry 6K SNP array v1 (Calle et al., 2018). For QTL mapping using FlexQTL^™^ (Bink et al., 2014), the genotyped SNPs of all plant materials were previously filtered. SNPs monomorphic in all populations, that had null alleles, with MAF < 0.05, with more than 5% of missing data, and showing errors in various genotypes were discarded. The selected markers were further checked by analyzing their genetic segregation using FlexQTL^™^ and by visual inspection when a double recombination event occurred within an interval smaller than 10 cM. A consensus genetic map from the selected SNPs was constructed. Those SNPs previously mapped in V×C (Calle et al., 2018) were assigned their genetic position. Those SNPs not previously mapped in the V×C map were integrated in the map by using their physical position on the peach genome v2.0.a1 (Verde et al., 2017).

QTL mapping for BT, each year and for BLUP values, using all the plant material, was carried out using a Bayesian multiple QTL model implemented in FlexQTL^™^ (Bink et al., 2014). In this work, only additive effects with normal prior distribution were considered. The models were set with a prior distribution of number of QTLs of 1 and 3 in order to assess sensitivity of posterior inference to the prior assumptions. Markov chain Monte Carlo (MCMC) simulation with minimum of 500,000 iterations for each prior number of QTL were performed until at least 100 effective chain samples (ECS) were reached for overall mean (*μ*), the residual variance ($\sigma_{e}^{2}$), the number of QTLs (*N_QTL_*), and the variance of QTLs (*_V_QTL*) (Gelman and Rubin, 1992; Sorensen and Gianola, 2002). A total of 1,000 samples (500,000 iterations with thinning value of 500) were stored for further posterior inferences. The inference in the number of QTLs was estimated using twice the natural log of Bayes factors (*2lnBF*) (Kass and Raftery, 1995) obtained after a pairwise comparison of models differing by one QTL from each other. Values of *2lnBF* higher than 2, 5, and 10 indicate positive, strong and decisive QTL evidences, respectively. Only QTLs with strong and decisive evidences were considered in this work. The percentage of variation explained (PVE) by each QTL was estimated as [(wVAt1/PV) × 100], where wVAt1 is the weighted variance and PV is the total phenotype variation (Mangandi et al., 2017). The genomic breeding value (GBV) for each individual and parent was predicted using QTL genotype probabilities, intensity and effect size (Bink et al., 2014).

### Haplotype Analysis

SNP haplotypes of the two most significant QTLs detected with an average 2lnBF higher than 10, were constructed for the parental cultivars and theirs ancestors. The haplotypes were designed to span the confidence interval with 2lnBF > 10 for these QTLs using BLUP model. The SNP haplotypes were estimated using SNP phase estimation of FlexQTL^™^ for all the cultivars, except for ‘Bing’ and ‘Napoleon’ that were estimated manually (for QTL on LG2) based on pedigree information and the availability of previously phased haplotypes (Cai et al., 2017). SNP haplotypes were also confirmed based on segregation. Mean phenotypic values of each of these QTL haplotypes and their combined effects were estimated in each segregating class of each population. Individual progenies with recombination events within these QTL regions were excluded from the analysis. For mean comparison of the phenotypic values within each population, Kruskal-Wallis, two-tailed Student’s t test and Tukey test (p < 0.01) post-hoc analysis were used. All statistical analysis were done using SPSS statistics v21.0.0 software (IBM, Chicago, IL, USA).

## Results

### Bloom Time Phenotyping, Segregation, and Heritability

**Figure 2:**
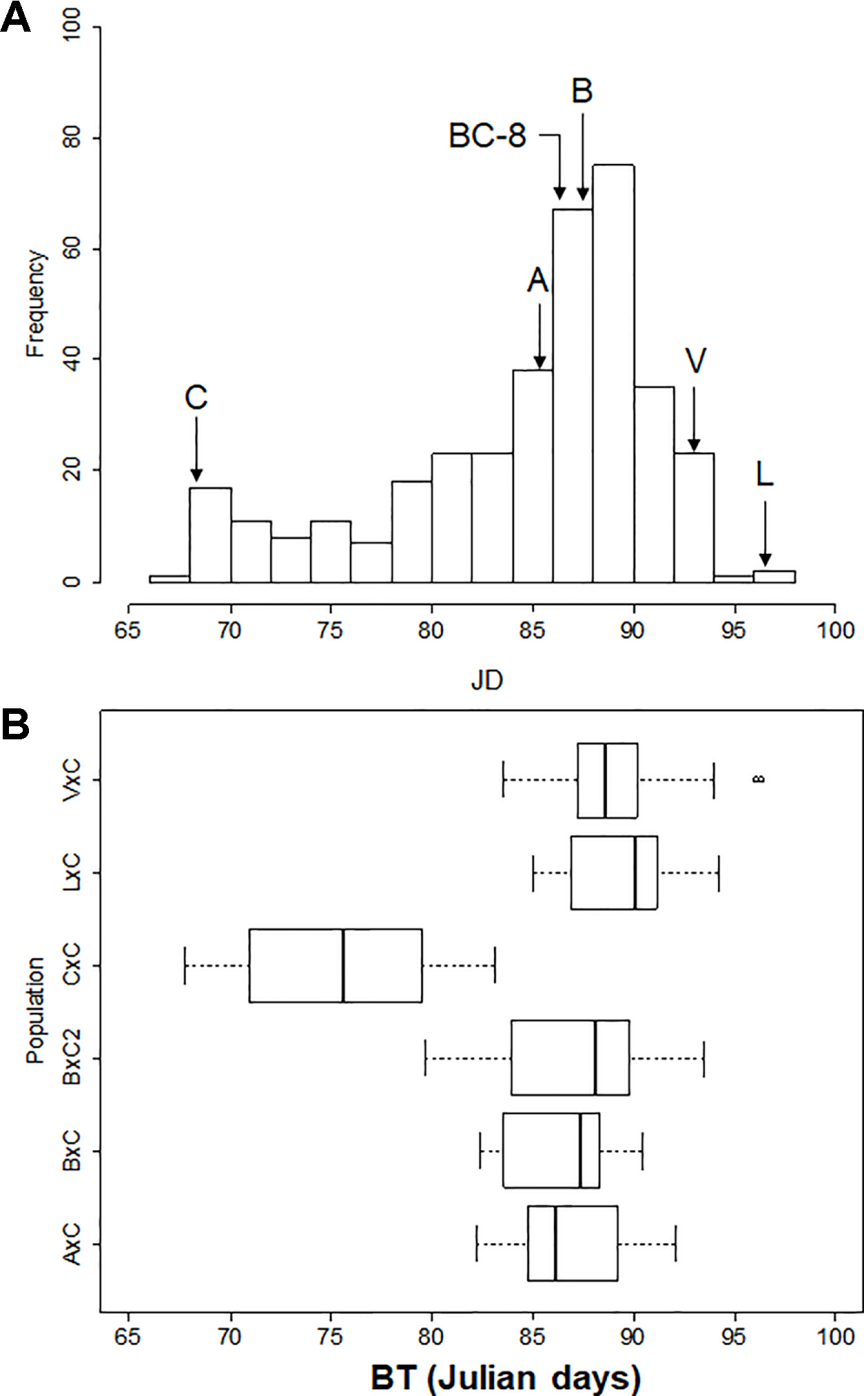
**(A)** Frequency distribution of bloom time BLUP values of plant materials analyzed. Letters with arrows indicate parents’ bloom times; ‘Ambrunés’ (A), ‘Brooks’ **(B)**, ‘B×C-08’ (BC-8), ‘Cristobalina’ (C), ‘Lambert’ (L) and ‘Vic’ (V). **(B)** Blooming period of each population (boxes). Bold line in the boxplot indicates the median value, the left and right sides of the box represent the 25^th^ and 75^th^ percentile, respectively. Whiskers represent the lower and upper extreme values.

BT was evaluated in the parental cultivars and populations during 4 consecutive years (**Figure 1**; **Supplementary Table S1**; **Supplementary Figure S1**). The parental cultivar ‘BC-8’ was phenotyped only the first year as the tree was in poor health in subsequent years. ‘Cristobalina’ was the earliest parental cultivar to bloom in all of the years [BLUP value: 69 JDs; **Figure 1** and **Figure 2**]. ‘Ambrunés’ ‘BC-8’ and ‘Brooks’ showed midseason flowering (**Figure 1** and **2**), while ‘Vic’ and ‘Lambert’ exhibited late blooming with BLUP values of 95 and 97 JDs, respectively (**Figure 1** and **2**). CR were fulfilled for ‘Cristobalina’ (550 h; Tabuenca, 1983) between mid-December to the first week of January during the 4 years of analysis (**Supplementary Figure S2**). The rest of the parental cultivars, ‘Ambrunés’ ‘Brooks,’ and ‘Lambert’ all had higher CR (900 to 1100 h; Tabuenca, 1983; Alburquerque et al., 2008). During the 4 years evaluated, the CR of these three cultivars were not fulfilled until mid-January to late February (**Supplementary Figure S2**).

The same blooming order (extra-early, mid, and late blooming) was observed for the parental cultivars each year, but differences in the BTs were observed between years (**Supplementary Table S1**; **Supplementary Figure S1**). BT was earliest in 2017 for the mid and late cultivars, which bloomed 16 to 17 days earlier than the average date of the rest of the years. However, for ‘Cristobalina’ the earliest bloom period occurred in 2016 (**Supplementary Table S1**; **Supplementary Figure S1**), while the latest bloom period for all parental cultivars occurred in 2018 (**Supplementary Table S1**; **Supplementary Figure S1**). In 2016, fulfilment of the CR and HR for ‘Cristobalina’ occurred early resulting in an early bloom. However, this was followed by a period of cold temperatures that delayed the flowering of the rest of the cultivars extending the blooming season (**Supplementary Figure S2**). In 2017, a high accumulation of chill hours during the early winter followed by a period of warmer temperatures in the beginning of February resulted in an earlier bloom for all the cultivars and a shorter blooming period (**Supplementary Figure S2**). In 2015 and 2018, although large amounts of chill were accumulated during the early winter, cold temperatures in February delayed bloom. Years 2017 and 2018 were colder and CR were fulfilled earlier in the year, but BT was earlier in 2016.

In the populations, different numbers of offspring were phenotyped each year, ranging from 258 (64%) in 2015 to 367 (90%) in 2018 (**Supplementary Table S1**). Only individuals (N = 360) with phenotypic data from two or more years were used to estimate BLUP values. The bloom period varied between years and populations, from 7 to 24 days. On average A×C, B×C, and L×C showed shorter bloom periods (10 to 12 days) than B×C F2, C×C and V×C (16 to 18 days) (**Supplementary Table S1**; **Supplementary Figure S1**). ‘Cristobalina’ self-pollination (C×C) was the earliest population to bloom (**Figure 1**and **Figure 2**), as on average, it bloomed 11 to 14 days earlier than the rest of populations (**Figure 1**and **Figure 2**). The mean BTs of A×C, B×C, and B×C F2 were similar, while V×C and L×C were the latest populations to bloom (**Figure 1**and **Figure 2**). Differences in the mean BT of the populations was observed between years. For all the populations, the earliest bloom period was in 2017 and latest in 2018 (**Supplementary Table S1**; **Supplementary Figure S1**). It was especially noticeable in 2017 when warm temperatures resulted in earlier flowering for all genotypes and the shortest bloom period (18 days) of all years. In 2016, warm temperatures in mid-February, resulted in very early bloom of some individuals from C×C population, but a cold period later on delayed the flowering of the rest of the population, resulting in the largest bloom period in all years evaluated.

BT distribution varied between populations and years. Only the smallest populations (B×C and L×C) fitted a normal distribution for all evaluated years and for BLUP values (Shapiro-Wilk test; Prob < W: 0.083-0.263). Populations B×C F2 and A×C fitted a normal distribution only two of the years (Shapiro-Wilk test; Prob < W: 0.085-0.664), whereas the remaining two populations (C×C and V×C), which are the largest, did not fit normal distributions in any of the years. BT of all the progenies together also did not show normal distribution for any of the years or BLUP values (**Figure 2**). In all cases, skewed distributions towards medium and late BT were observed. In fact, only some C×C offspring were extra-early blooming, and only some B×C F2 offspring were early blooming. The rest of the plant materials were medium to late blooming, even though all populations (except B×C F2) had ‘Cristobalina’ (extra-early blooming) as one of the parental cultivar. Transgressive segregation toward late blooming was also observed in the 4 years and for BLUP values for all populations except L×C. On the other side, transgressive segregation towards early blooming was only observed in the self-pollination populations (C×C and B×C F2; **Figure 2**; **Supplementary Table S1**; **Supplementary Figure S1**).

Highly significant (p < 0.01) and positive correlations were observed for BTs between years and BLUP value (r = 0.897 to 0.966; **Supplementary Table S2**). BT broad-sense heritability (*H^2^*) estimated for all populations together was 0.97, and for each population individually, the *H^2^* was also high, with values ranging from 0.85 to 0.96 (**Supplementary Table S1**).

### QTL Analysis

Quality filtering of the SNP markers resulted in 1,269 (22.3%) SNPs selected for map construction (**Supplementary Tables S3** and **S4** ) and QTL analyses. These selected SNPs covered a total genetic length of 721.98 cM with an average marker density of 0.57 cM (1 SNP per 176 kb) and a maximum gap between markers of 10.95 cM (1.43 Mbps) located on LG 7 (**Supplementary Table S4**).

**Figure 3:**
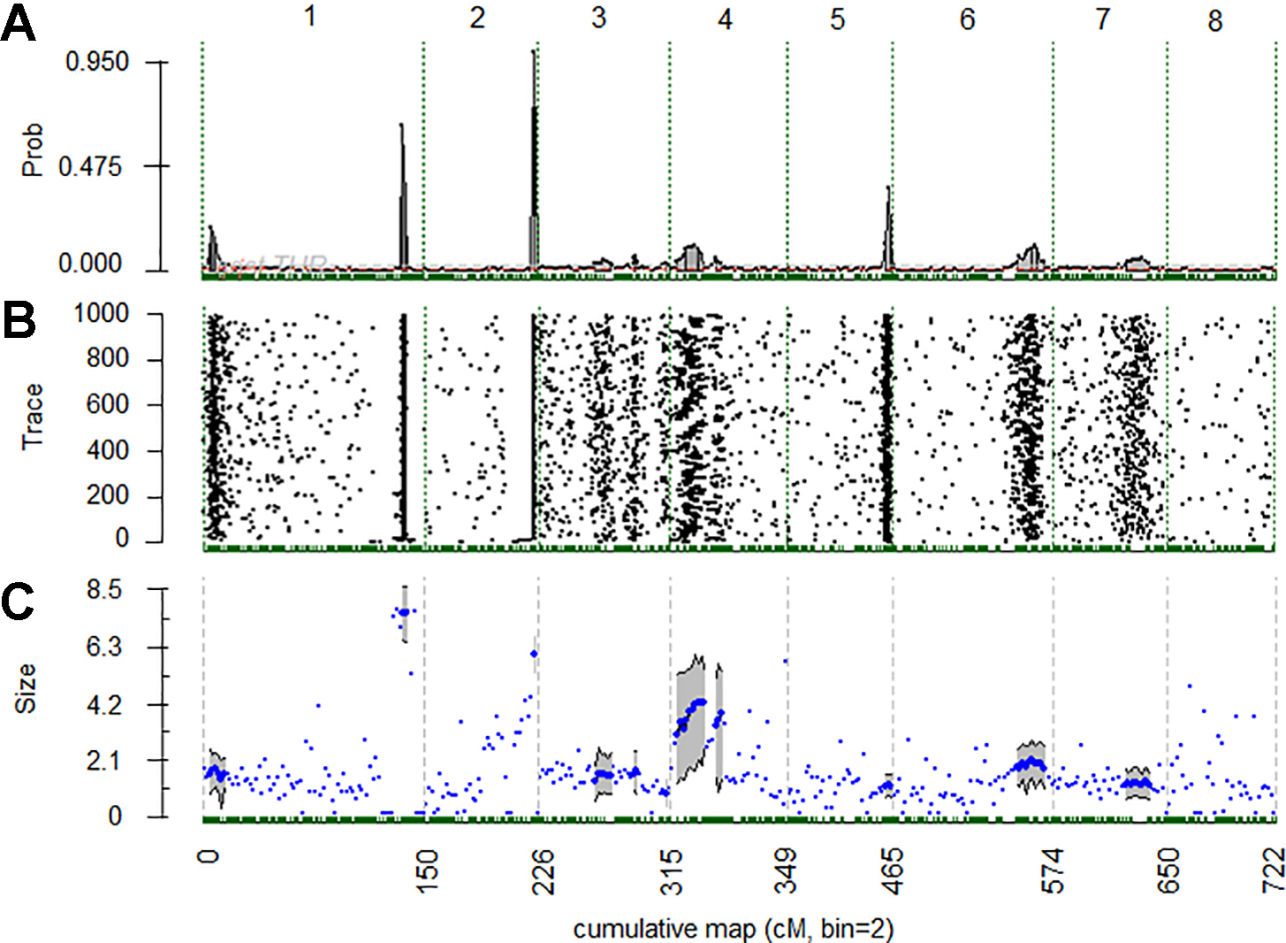
**(A)** Posterior probabilities of quantitative trait loci (QTL) positions along the genome with a 2 cM bin resolution. **(B)** Trace plot of Markov chain Monte Carlo samples for QTL position. **(C)** Posterior mean (blue dots) and 90% credible region (gray shade) for estimate additive QTL effects for chromosomal regions of 2 cM bins with positive evidence (2lnBF10 > 2) for QTL presence. Vertical dashed lines identify the starts and ends of chromosomes.

QTL analysis using BLUP values of the 4 years in all the populations revealed three significant BT QTLs located on LGs 1, 2 and 4, of which *qP-BT1.1^m^* and *qP-BT2.1^m^* were found with decisive evidences (2lnBF > 10) (**Table 1** and **Figure 3**). QTL on LG1, *qP-BT1.1^m^*, explained the largest percentage of phenotypic variation (PVE; 32.4%) and was associated with a mean additive effect of 7.4 days (**Table 1** and **Figure 3**). The other decisive QTL, *qP-BT2.1^m^*, had a PVE of 15.2% and an additive effect of 5.9 days (**Table 1**). Also a QTL on LG4 (*qP-BT4.1^m^*) with strong evidence was identified, although it had a lower additive effect (3.6 days) and PVE (6.0%) than the other two major QTLs (**Table 1** and **Figure 3**).

**Table 1:** Bloom time quantitative trait loci (QTLs) identified with strong evidence (2lnBF > 5) in multiyear analysis [best linear unbiased prediction (BLUP) values] and single year analyses. Bold indicates decisive evidence for a QTL (2lnBF > 10).

Year	QTL	LG^1^	cM^2^	QTL peak (cM)	Physical position^3^ (Mbp)	Max 2lnBF	Average 2lnBF	Mean Additive effect	PVE (%)
**BLUP**	***qP-BT1.1^m^***	**1**	**137-139**	**137**	**43.28 - 43.54**	**10.9**	**9.5**	**7.4**	**32.4**
**BLUP**	***qP-BT2.1^m^***	**2**	**73-75**	**75**	**26.96 - 29.68**	**18.8**	**18.8**	**5.9**	**15.2**
BLUP	*qP-BT4.1^m^*	4	4-23	19	0.92 - 5.44	5.1	4.6	3.6	6.0
**2015**	***qP-BT1.1^m^***	**1**	**137-141**	**139**	**43.28-44.09**	**12.2**	**9.8**	**9.6**	**57.6**
**2015**	***qP-BT2.1^m^***	**2**	**73-75**	**75**	**26.96-29.68**	**19.6**	**11.8**	**5.1**	**15.0**
**2016**	***qP-BT1.1^m^***	**1**	**137-147**	**139**	**43.28-46.10**	**9.6**	**7.3**	**7.3**	**14.2**
**2016**	***qP-BT2.1^m^***	**2**	**73-75**	**75**	**26.96-29.68**	**32.8**	**32.7**	**10.8**	**23.0**
2016	*qP-BT7.1*	7	55-61	57	17.40-18.88	5.7	5.4	6.6	3.9
**2017**	***qP-BT1.1^m^***	**1**	**133-139**	**137**	**42.77-43.53**	**12.3**	**9.0**	**7.3**	**25.6**
**2017**	***qP-BT2.1^m^***	**2**	**73-75**	**75**	**26.96-29.68**	**16.7**	**11.6**	**3.0**	**15.7**
2017	*qP-BT4.2*	4	31-49	39	6.71-10.24	8.4	6.5	2.7	4.1
2017	*qP-BT5.1*	5	54-71	69	13.08-18.41	9.0	6.3	1.0	0.4
2017	*qP-BT7.1*	7	39-61	51	15.34-18.88	6.8	5.9	1.1	1.4
2018	*qP-BT1.2*	1	5-21	7	2.11-6.42	9.6	6.1	1.6	2.4
**2018**	***qP-BT1.1^m^***	**1**	**133-139**	**135**	**42.77-46.10**	**10.6**	**9.1**	**7.6**	**60.9**
**2018**	***qP-BT2.1^m^***	**2**	**73-75**	**75**	**26.96-29.68**	**19.4**	**19.4**	**5.5**	**23.0**
2018	*qP-BT4.2*	4	33-37	35	6.82-7.83	6.3	5.9	3.1	3.2
**2018**	***qP-BT5.1***	**5**	**67-71**	**69**	**16.78-18.41**	**12.1**	**10.2**	**1.4**	**2.5**
2018	*qP-BT7.1*	7	37-57	51	14.84-18.16	7.1	6.1	1.3	1.5

^1^LG: Linkage group. ^2^cM: centiMorgan. ^3^: Physical position on Peach Genome v2.0.a1 (Verde et al., 2017).

In the QTL analyses for individual years, the two major QTLs detected on LGs 1 and 2 in the 4-year analysis were also detected every single year with strong or decisive evidence (**Table 1**). In these years, the PVE of *qP-BT1.1^m^* and *qP-BT2.1^m^* ranged from 14.2% to 60.9%, and from 15.0% to 23.0%, respectively. The lowest and highest PVE for *qP-BT1.1^m^* were observed in 2016 and 2018, respectively, while the mean additive effect ranged from 7 to 10 days between 2016 and 2015, respectively. For *qP-BT2.1^m^*, the lowest and highest PVE were observed in 2015 and 2016, respectively, and the mean additive effect ranged from 3 to 11 days in 2017 and 2016, respectively. In 2016, the PVE and mean additive effect exhibited by *qP-BT2.1^m^* was larger than that observed by *qP-BT1.1^m^* (**Table 1**). In other years, like 2017 and 2018, in which the effects of these two QTLs were lower, additional QTLs with minor effects were detected.

Minor QTLs were detected on LGs 1, 4, 5, and 7 (*qP-BT1.2*, *qP-BT4.2*, *qP-BT5.1*, *qP-BT7.1*; **Table 1**). The significance of these QTLs varied between years, with *qP-BT1.2* detected only in 2018, *qP-BT4.2* detected in 2017 and 2018, *qP-BT5.1* detected in 2017 and 2018, and *qP-BT7.1* detected in 2016, 2017 and 2018. The QTL on LG5, *qP-BT5.1,* was most significant in 2018, showing decisive evidence but none of these minor QTL showed PVE higher than 5%. Nevertheless, *qP-BT7.1,* revealed an additive effect of up to 7 days in 2016.

### Estimation of QTL Genotypes and Genomic Breeding Value

**Figure 4:**
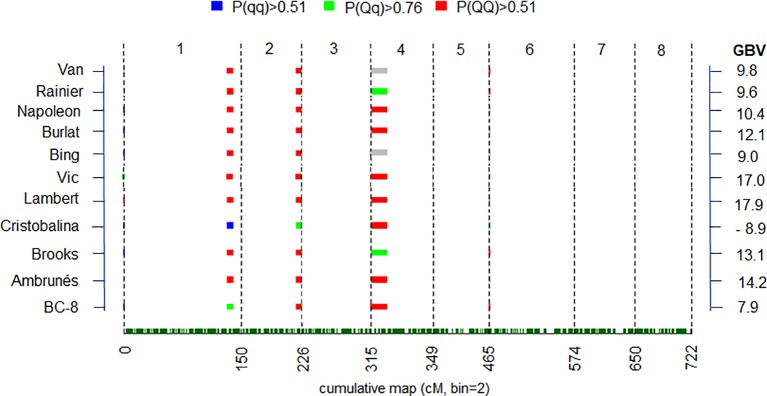
Posterior estimates of parental quantitative trait loci (QTL) genotype probabilities in QTL regions with strong and decisive QTL evidences (2lnBF > 5) for best linear unbiased prediction (BLUP) values. Red, green and blue colors represent positive evidence for QTL genotypes QQ, Qq, and qq genotypes, respectevely. “Q” and “q” denote alleles with high and low phenotype values, respectively. Gray colors indicate unclear genotype estimation. Genome breeding value (GBV) for each cultivar is indicated at right.

QTL genotype estimation was carried out for QTL regions with either strong or decisive evidence using BLUP value (**Figure 4**) for the parental cultivars and the ancestors in the collection (**Figure 4**). For the major QTLs on LG1 (*qP-BT1.1^m^*) and LG2 (*qP-BT2.1^m^*), ‘Cristobalina’ was predicted to be homozygous for alleles associated with early bloom (*qq* = low phenotype value) for the LG1 QTL and predicted to be heterozygous (*Qq*) for the LG2 QTL (**Figure 4**). ‘BC-8’ an offspring from the cross of ‘Brooks’ and ‘Cristobalina’ was heterozygous for the early bloom allele for the LG1 QTL (*qP-BT1.1^m^*). No other parental cultivar was predicted to have early BT alleles for these two QTL, instead the remaining parental cultivars were predicted to be homozygous (*QQ*) for LG1 and LG2 QTL alleles for later BT (**Figure 4**). This indicates that ‘Cristobalina’ is the only cultivar that contributed early BT alleles for the major QTLs *qP-BT1.1^m^* and *qP-BT2.1^m^* of all the plant materials. For the QTL on LG4 (*qP-BT4.1^m^*), only ‘Rainier’ and its offspring ‘Brooks’ were predicted to be heterozygous for early BT alleles, while the rest, including ‘Cristobalina’ were predicted to be homozygous (*QQ*) for late bloom alleles.

Differences in the predicted genotypes were used to estimate genome breeding value (GBV) of parents and ancestors (**Figure 4**). Differences of as much as 27 JDs (almost 1 month) were observed between the GBV of the earliest and latest blooming cultivars (**Figure 4**). All but ‘Cristobalina’ had GBV associated with delayed flowering. ‘Cristobalina’ had the lowest GVB, due to the relative abundance of alleles predicted to result in earlier flowering of 8.9 JDs. In contrast, ‘Vic’ and ‘Lambert’ cultivars had the GBV most associated with late flowering (17.0 and 17.9 JDs, respectively; **Figure 4**).

### QTL Haplotype and Genotype Analysis

**Figure 5:**
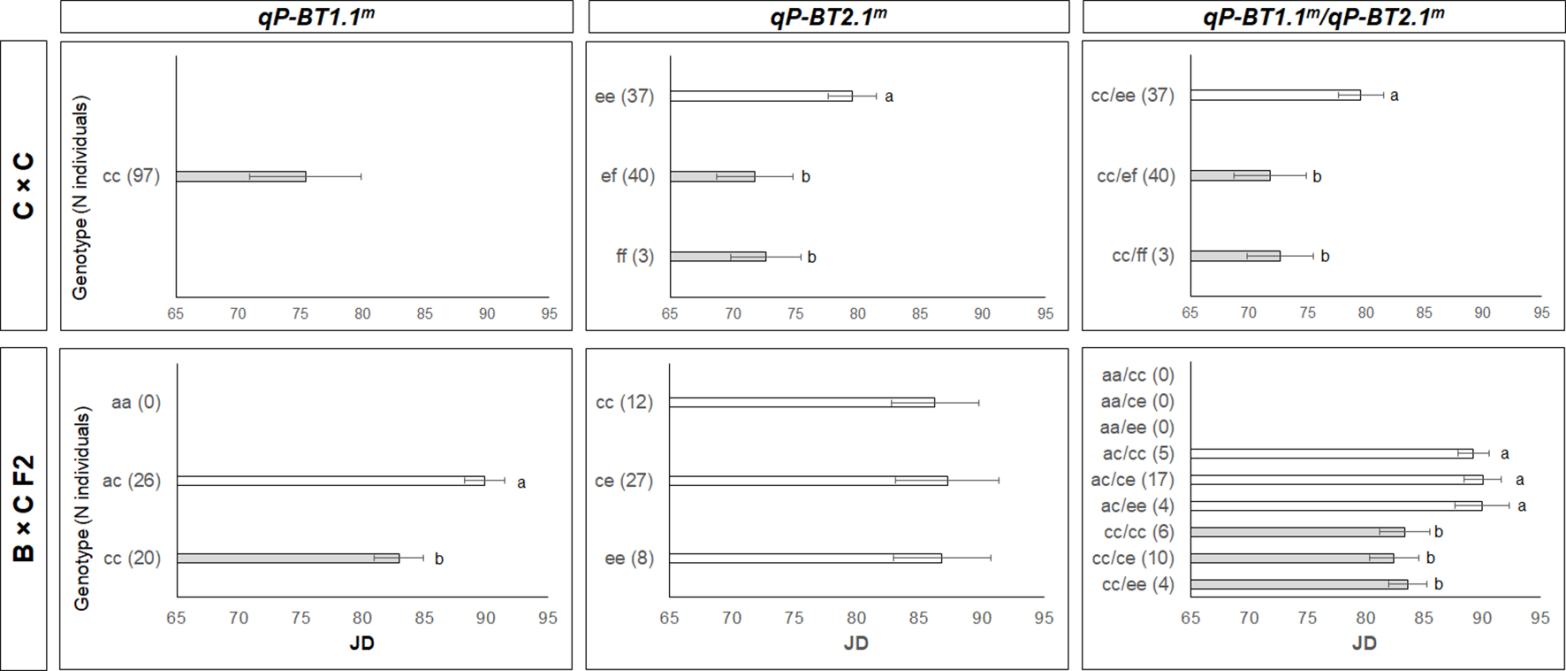
Bloom time (BT) mean values for offspring based on the haplotypes for the major quantitative trait loci (QTLs) detected on LG1 (qP-BT1.1m) and LG2 (qP-BT2.1m) individually and the two QTLs together. The offspring are from the two F2 populations (C×C and B×C F2). Significant differences between genotypes are indicated by different letters (P < 0.05).

The haplotypes (alleles) of the two most significant QTLs detected, those on LG1 (*qP-BT1.1^m^*) and LG2 (*qP-BT2.1^m^*), were constructed for the parental cultivars and their ancestors (**Supplementary Table S5**) to investigate their phenotypic effects (**Figure 5**). Haplotypes of *qP-BT1.1^m^* and *qP-BT2.1^m^* were constructed with 4 and 14 SNPs, respectively (**Supplementary Table S5**). These intervals spanned physical regions of 0.26 and 2.70 Mbps, respectively. The haplotypes for *qP-BT1.1^m^* were constructed for the region of chromosome 1 between 43.28 to 43.54 Mbp, while the haplotypes for *qP-BT2.1^m^* covered the chromosome 2 interval region of 26.98 to 29.68 Mbp (Peach genome v2.0.a1). For *qP-BT1.1^m^*, three haplotypes, *H1-a* to *H1-c* were identified (**Supplementary Table S5**). *H1-c* was only found in ‘Cristobalina’ (homozygous for this allele) and in the selection ‘BC-8’ (heterozygous). The remaining two haplotypes, *H1-a* and *–b,* were found in the rest of cultivars (**Supplementary Table S5**). For the QTL *qP-BT2.1^m^*, 10 haplotypes were identified (*H2-a* to *H2-j*). All the parental cultivars had unique haplotypes except ‘Brooks’ and ‘Lambert’ that shared *H2-d*, and ‘BC-8’ that shares haplotypes with its parental cultivars, ‘Brooks’ and ‘Cristobalina’ (**Supplementary Table S5**).

The estimation of the mean phenotypic values of these QTL haplotypes in the F_2_ populations (C×C and B×C F2) revealed that for the QTL *qP-BT1.1^m^*, those individuals that were homozygous for *H1-c*, like ‘Cristobalina’ (*cc*), showed the earliest BTs (**Figure 5**). As in C×C, this QTL was not segregating and all the progeny were also ‘*cc*,’ with a mean BT of 75 JDs. In B×C F2, this QTL was segregating but segregation distortion was observed as no ‘*aa*’ individuals were identified. In the two remaining segregating classes, individuals with the ‘*cc*’ genotype showed a mean difference of almost 7 JDs earlier blooming that those with ‘*ac*’ genotype (**Figure 5**).

For the QTL *qP-BT2.1^m^*, *H2-f* was associated with early flowering. In C×C (‘Cristobalina’; ‘*ef*’), this QTL segregated in three classes, with offspring that were ‘*ef*’ and ‘*ff*’ flowering on average 7 JDs earlier that those that were ‘*ee*’ (**Figure 5**). As no significant differences were observed between these two classes, *H2-f* appeared to be dominant to *H2-e* (**Figure 5**). B×C F2 also segregated in three classes for this QTL, but no significant differences were observed among them (**Figure 5**). Since *H2-f* was not inherited in ‘BC-8’ (*ce*), the effect of this haplotype could not be investigated in this population.

The interaction of these two major QTLs showed that those individuals homozygous for *H1-c* were the earliest to bloom for both populations. Within these, those that also had *H2-f* showed the earliest BT (**Figure 5**).

In the F_1_ populations (**Supplementary Figure S3**), for *qP-BT1.1^m^*, “*ac*” genotypes were always earlier blooming (approx. 2 to 3 days that those that are “*bc*”; **Supplementary Figure S3**), indicating that *H1-a* was associated with earlier BT compared to *H1-b*. For *qP-BT2.1^m^*, genotypes with *H2-f* showed earlier BT (1 to 7 JDs) compared to individuals without it. In addition, segregation distortion against *H2-f* was observed in all the populations, being most evident in B×C, as none of the progeny had this haplotype (**Supplementary Figure S3**). A genotype interaction of both QTLs in the F_1_ populations was identified as individuals heterozygous for *H1-a*/*H2-f*, showed earlier BT than those individuals with other genotype combinations (**Supplementary Figure S3**).

## Discussion

‘Cristobalina’ the earliest blooming parental cultivar, has a low CR (176-550 h under 7°C; Tabuenca, 1983; Alburquerque et al., 2008); which is consistent with its native origin close to the Mediterranean coast in eastern Spain (Herrero, 1964). Plant’s CR are typically correlated to the climate in the area of origin (Abbott et al., 2015). In our experimental location, which experiences a higher chilling accumulation, it is likely that the early blooming of ‘Cristobalina’ is due to its low CR, as earlier blooming has been observed in cultivars with lower CR (Alburquerque et al., 2008; Castède et al., 2014). ‘Cristobalina’ is also self-compatible (Wünsch and Hormaza, 2004) due to a single mutation affecting pollen tube growth (Cachi and Wünsch, 2011; Ono et al., 2018). Natural self-compatible mutations are rare in sweet cherry; however, this mutation may be especially beneficial in this low chill cultivar because mating partners with overlapping flowering times would be scarce (Cachi et al., 2014).

The same BT (JD) order, early, mid- and late bloom, of the parental cultivars in the 4 years, independently of the year temperatures, confirms the genetic determination of this trait. As previously demonstrated, BT in cherries is a quantitative trait with high heritability (Dirlewanger et al., 2012; Castède et al., 2014; Cai et al., 2018). High heritability values for this trait were also observed in this work for the 4 years (0.85 to 0.96), and these values are in the same range as those estimated previously for sweet and sour cherry (0.88 to 0.96; Wang et al., 2000; Dirlewanger et al., 2012; Castède et al., 2014; Cai et al., 2018; Piaskowski et al., 2018). However, BT differences between years are highly dependent on environmental conditions and how these conditions impact CR fulfillment. For example, the coldest winters did not result in the earliest BT.

Within the populations, only individuals from F_2_ populations (C×C and B×C F2) showed transgressive segregation towards early blooming, whereas F_1_ populations showed transgressive segregation and skewed distribution towards late flowering, revealing possible dominance of the late bloom alleles in this plant material. In sweet cherry, skewed segregation in F_1_ populations towards high CR, but not late bloom, was also observed (Castède et al., 2014). This was also the case in almond, where *Late bloom* (*Lb*) is dominant (Ballester et al., 2001), and in Japanese plum and apricot F_1_ populations from low CR cultivars (Campoy et al., 2011; Salazar et al., 2016; Kitamura et al., 2018). However, transgressive segregation toward both early and late blooming was observed in peach F_2_ populations (Fan et al., 2010; Bielenberg et al., 2015). The effect of (recessive) alleles in the homozygous state will be possible to detect in F_2_ populations (like C×C and B×C F2), which may explain why transgressive segregation towards early bloom in our plant material is only observed in the F_2_ populations. The extended bloom period observed in the larger populations (C×C, V×C) may also have resulted from additional climatic variation experienced during this longer BT duration. This effect was also observed by Castède et al. (2014).

### Two Major BT QTLs on LG1 (*qP-BT1.1^m^*) and LG2 (*qP-BT2.1^m^*)

The major QTL identified on LG1 (*qP-BT1.1^m^*) has been previously detected in sweet cherry (Dirlewanger et al., 2012; Castède et al., 2014). However, in these works, the variation explained and additive effect of this QTL were lower than observed herein. Dirlewanger et al. (2012) first identified this LG1 QTL in ‘Lapins’ with a PVE ranging from 9.3 to 17.5% and an additive effect of 2.5 days. Castède et al. (2014) reported the same QTL region for two of three years for a ‘Regina’ × ‘Garnet’ population, with similar additive effect (1.4 days) and mean PVE (8%). In our work, this QTL represented 32.4 of PVE and has an additive effect of 7.4 JDs. These results indicate that BT of our plant material, in our environmental conditions, was determined by this QTL in a larger proportion than in earlier works in sweet cherry.

A CR QTL overlapping with this LG1 BT QTL was also identified in sweet cherry (Castède et al., 2014), confirming the correlation between both traits and the relevance of CR for BT in the species. This BT and CR QTL on LG1 has also been described in other *Prunus* species like peach, apricot and almond, as a main QTL controlling these traits (Olukolu et al., 2009; Fan et al., 2010; Dirlewanger et al., 2012; Sánchez-Pérez et al., 2012; Salazar et al., 2013; Zhebentyayeva et al., 2014; Romeu et al., 2014; Bielenberg et al., 2015). In our work, the high significance and effect of this QTL indicates that, in our conditions, the BT of the plant material analyzed is probably more dependent on CR than other materials analyzed in different environments.

Candidate genes of the LG1 QTL have been described in peach and sweet cherry (Bielenberg et al., 2008; Fan et al., 2010; Castède et al., 2015). The position of this QTL overlaps with the region where six *DORMANCY ASSOCIATED MADS-box* (*DAM1-6*) genes have been identified as major genes controlling CR and BT in peach (Bielenberg et al., 2008; Fan et al., 2010). In the *evergrowing* peach mutant, that lacks response to winter cold, four of these genes are deleted and the other two are not expressed (Bielenberg et al., 2008). Castède et al. (2015) mapped two of these *DAM* genes (*DAM5* and *DAM6*) within the interval of this QTL in ‘Lapins’ sweet cherry. It is likely that the ‘Cristobalina’ alleles of these genes are contributing to low CR and early blooming, and the large effect of this QTL in the plant material analyzed. ‘Cristobalina’ contributed *H1-c* for this QTL, which was associated with earlier flowering, as BT is earliest (7 days) when *H1-c* is homozygous, as is the case for ‘Cristobalina’ and in all the C×C population. Previously, a large amount of homozygosity was observed in ‘Cristobalina’ and therefore also in the self-pollinated population (Calle et al., 2018). More specifically, a large homozygous region at the bottom of LG1 (26.23 to 47.81 Mbp), overlapping with this BT QTL was observed (Calle et al., 2018). A smaller difference (approx. 2 to 3 days) observed between the two remaining haplotypes (*H1-a, -b)* is in agreement with the finding that this QTL was detected at lower PVE in other works (Dirlewanger et al., 2012; Castède et al., 2014), where the allele *H1-c* was probably not present.

The second major QTL was identified on LG2 (*qP-BT2.1^m^*). This QTL also overlaps with a CR and BT QTL previously described in sweet cherry (Castède et al., 2014). The PVE and additive effect of this QTL in previous work (3.6%–6.5% PVE; 0.8–2.8 day; Castède et al., 2014) was also lower than observed in our study (12.8-15.2%; 5.5 JDs). This QTL has also been identified in apricot and in the interspecific cross of peach and *P. davidiana*, but explained a lower PVE than herein (Quilot et al., 2004; Olukolu et al., 2009; Dirlewanger et al., 2012). *SOC1*, a MADS-box gene, has been identified as a strong candidate gene for CR and BT underlying this QTL in sweet cherry and apricot (Trainin et al., 2013; Castède et al., 2015). However, the physical position of this gene (Castède et al., 2015) is not within the interval of the QTL detected in this work. Among other candidate genes identified in this region in sweet cherry (Castède et al., 2015), only the candidate gene, *FAR-RED IMPAIRED RESPONSE 1* (*FAR1*) is within the interval of this QTL in our work*. FAR1* has been described as a negative regulator of seasonal growth and flowering time in *Arabidopsis* and the loss of function of this gene resulted in plants with early flowering (Ritter et al., 2018). Therefore, this gene seems a good candidate gene for BT regulation in the genus and further work to characterize this gene in this plant material is ongoing. A larger number of haplotypes (10) were detected for this QTL, maybe due to the haplotypes being constructed across a larger genomic region, and only *H2-f* from ‘Cristobalina’ was shown to associate with earlier bloom (7 days). As observed for the QTL detected on LG1, ‘Cristobalina’ alleles for the underlying genes are likely responsible for the higher effect of this QTL in this plant material.

Segregation distortion was observed for some populations in both major QTLs on LGs 1 and 2. Segregation distortion in these genomic regions was previously detected in these populations (Calle et al., 2018) and in other *Prunus* species (Fan et al., 2010; Bielenberg et al., 2015). This distortion may be associated with segregation of lethal recessives alleles. However, since a relationship between seed and bud dormancy control has been reported (Leida et al., 2012; Abbot et al., 2015; Wang et al., 2016), it is possible that differences in seed dormancy may have affected seed germination and survival resulting in segregation distortion.

### Other Minor QTLs

A minor effect QTL identified by BLUP values in this work was located on LG4. This QTL (*qP-BT4.1^m^*) has also been previously detected in cherries (Dirlewanger et al., 2012; Castède et al., 2014; Cai et al., 2018) and other *Prunus* species (Fan et al., 2010; Sánchez-Pérez et al., 2012; Zhebentyayeva et al., 2014; Bielenberg et al., 2015; Kitamura et al., 2018). This QTL has been reported as the major QTL controlling CR and BT (17.5% to 47.2% PVE) in sweet (Dirlewanger et al., 2012; Castède et al., 2014) and sour cherry (Cai et al., 2018), almond (Sanchez-Pérez et al., 2012) and Japanese apricot (Kitamura et al., 2018). However in our work, this QTL explained a smaller part of the variation (6.0%) (**Table 1**) and was not detected all years. Several works indicated that the LG4 QTL had a larger effect on BT of high chill cultivars (Castède et al., 2014; Kitamura et al., 2018), while in low chill cultivars, as in this work, the variation in BT is more dependent in the QTL on LG1 (Fan et al., 2010; Sánchez-Pérez et al., 2012; Zhebentyayeva et al., 2014; Salazar et al., 2016). LG1 candidate genes (*DAM1-6*) are related to CR, and therefore these genes may have a larger contribution to BT of low chilling cultivars. In contrast, BT for high CR cultivars may be less dependent on CR, and the underlying gene(s) for the LG4 QTL has yet to be determined (Zhebentyayeva et al., 2014; Castède et al., 2015).

Three additional minor QTLs were detected in single year analyses, *qP-BT1.2, qP-BT5.1,* and *qP-BT7.1.* The QTLs on LGs 2 and 7, *qP-BT1.2* and *qP-BT7.1,* were previously detected in sweet cherry also with small effects (Dirlewanger et al., 2012; Castède et al., 2014), but in this work it is shown that in certain environmental conditions, like those in 2016, *qP-BT7.1,* may have a large effect. The QTL on LG5, *qP-BT5.1*, has not been previously reported in sweet or sour cherry, but it has been described in the same genomic region in peach (Bielenberg et al., 2015). ‘Cristobalina’ was the only cultivar in this work which is heterozygous for this region (data not shown), and thus the identification of this QTL was probably due to the presence of this cultivar, and is probably associated with a rare allele found in ‘Cristobalina.’

### Breeding and Genome Breeding Value

The predicted genotypes for the QTL identified were used to calculate breeding value. This estimation for the parental and ancestor cultivars studied offers powerful information for breeding with these cultivars. ‘Cristobalina’ can be used for breeding for low CR cultivars as this work shows it is the only evaluated cultivar that exhibited early flowering due to the presence of early bloom and low chill requirements alleles in the two major QTLs affecting these traits. A similar situation was observed in peach (Hernández Mora et al., 2017), where the lowest breeding values correlated with early flowering were identified in peach landraces. This highlights the benefits of introducing exotic germplasm in breeding programs to widen the range of trait variation. Specifically for breeding for low CR cultivars with ‘Cristobalina’ selecting for *H1-c* and *H2-f* from QTLs *qP-BT1.1^m^* and *qP-BT2.1^m^*, respectively, is predicted to result in earlier blooming offspring. However, recovery of both haplotypes (*H1-c*/*H2-f*) together, may require a large number of progeny, as segregation distortion against the earlier haplotype *H2-f* was observed. At the same time, embryo rescue and *in vitro* embryo culture may be required to obtain low chilling descendants from crosses with ‘Cristobalina’ as the maternal parent.

If breeding for late blooming, allele *H1-b* rather than *H1-a,* should be selected for the QTL on LG1. As this QTL interval has been much narrowed in this work, and a good representation of sweet cherry breeding founders and parental cultivars is included herein, this information will also be useful for sweet cherry breeding of other plant material that do not include ‘Cristobalina’. For the QTL on LG2, no conclusive evidence of late blooming haplotypes that were sufficiently predictive to be used in breeding recommendations were observed for the haplotypes detected in the parental and ancestor cultivars. For the QTL on LG4 that had a minor effect in this work, but high effect in other plant material with higher CR, selecting offspring from cultivars such as ‘Rainier’ and ‘Brooks,’ which are heterozygous for early and late bloom alleles in this QTL, would be useful for introducing an earlier allele.

## Conclusions

Multi-year analysis of six pedigree-linked populations from different genetic backgrounds that descended from the low chilling ‘Cristobalina,’ resulted in the identification of robust BT QTLs for this highly heritable trait. Two major QTLs located on LGs 1 and 2 were identified that explained 47.6% of total phenotype variation. This work, representing the first genetic analysis of F_2_ populations in sweet cherry and possible with the self-compatible ‘Cristobalina,’ was instrumental to characterizing the haplotype effects of these QTLs. Since the B×C F2 population resulted from self-pollination, it was possible to compare the effect of homozygous alleles in ‘Cristobalina’ not segregating in the other F_1_ populations. BT is an essential component of cultivar adaptation to low-chill growing conditions and this trait is currently of high interest to breeders to extend sweet cherry growing to warmer areas. The identification and characterization of the haplotypes of these QTLs will enable marker-assisted breeding for this trait. The discovery of the major QTL on LG1 is consistent with the *DAM* gene(s) as the CR determinant in *Prunus,* and further suggests that ‘Cristobalina’ is homozygous for a unique early mutant of one or more of the *DAM* genes. Further work is ongoing to characterize these genes in this plant material.

## Data Availability Statement

The datasets generated for this study can be found in the Genome database for Rosaceae, https://www.rosaceae.org/accession https://www.rosaceae.org/accession number tfGDR1040.

## Author Contributions

AC carried out phenotyping, data analysis and interpretation, and manuscript writing. LC advised on QTL analysis and interpretation. LC, AI, and AW participated in experimental and data analysis supervision and design and in manuscript revision. AW was also a major contributor in manuscript writing. All authors read and approved the final manuscript.

## Funding

This work and its publication was funded by Spanish Government “Ministerio de Economía Industria y Competitividad,” “Agencia Estatal de Investigación” (AEI), and “Instituto Nacional de Investigación y Tecnología Agraria y Alimentiaria (INIA) by research projects RTA2015-00027-00-00, and RFP2015-00015-00-00, and FEDER funds; and by “Grupo de Investigación de la Comunidad de Aragón” A12-17R (“Fruticultura. Caracterización, Adaptación y Mejora Genética”) of “Departamento de Innovación, Investigación y Universidad,” “Gobierno de Aragón.” AC was funded by “Departamento de Innovación, Investigación y Universidad,” “Gobierno de Aragon” by PhD programme “Subvenciones destinadas a la contratación de personal investigador en formación 2015-2019.” LC was supported by the USDA-NIFA-Specialty Crop Research Initiative project, RosBREED: Enabling marker-assisted breeding in Rosaceae (2009-51181-05808) and RosBREED 2: Combining disease resistance with horticultural quality in new rosaceous cultivars (2014-51181-22378).

## Conflict of Interest

The authors declare that the research was conducted in the absence of any commercial or financial relationships that could be construed as a potential conflict of interest.

## Abbreviations

BF, Bayes factor; BLUP, Best linear unbiased prediction; BT, Bloom time; JD, Julian day; CR, Chilling requirements; DAM, Dormancy associated MADs-box; EVG, Evergrowing peach mutant; FAR1, Far-red impaired response 1; GBV, Genome breeding value; H2, Broad-sense heritability; HR, Heat requirements; LG, Linkage group; MAS, Marker-assisted selection; PVE, Percentage of variation explained; QTL, Quantitative trait locus; SNP, Single nucleotide polymorphism.

## References

[B1] Abbott A. G. Zhebentyayeva T. Barakat A. Liu Z. A. (2015). The genetic control of bud-break in trees. Adv. Bot. Res. 74, 201–228. 10.1016/bs.abr.2015.04.002

[B2] Alburquerque N. García-Montiel F. Carrillo A. Burgos L. (2008). Chilling and heat requirements of sweet cherry cultivars and the relationship between altitude and the probability of satisfying the chill requirements. Env. Exp. Bot. 64 (2), 162–170. 10.1016/j.envexpbot.2008.01.003

[B3] Allard A. Bink M. C. Martinez S. Kelner J. J. Legave J. M. di Guardo M. (2016). Detecting QTLs and putative candidate genes involved in budbreak and flowering time in apple multiparental population. J. Exp. Bot. 67 (9), 2875–2888. 10.1093/jxb/erw130 27034326 PMC4861029

[B4] Anciro A. Mangandi J. Verma S. Peres N. Whitaker V. M. Lee S. (2018). FaRCg1: a quantitative trait locus conferring resistance to *Colletotrichum* crown rot caused by *Colletotrichum gloeosporioides* in octoploid strawberry. Theo. App. Genet. 131, 2167–2177. 10.1007/s00122-018-3145-z 30032317

[B5] Anderson J. L. Seeley S. D. (1993). Bloom delay in deciduous fruits. Hortic. Rev. 15, 97–144. 10.1002/9780470650547.ch3

[B6] Atkinson C. J. Brennan R. M. Jones H. G. (2013). Declining chilling and its impact on temperate perennial crops. Env. Exp. Bot. 91, 48–62. 10.1016/j.envexpbot.2013.02.004

[B7] Baggiolini M. (1980). “Stades repères du cerisier - Stades repères du prunier. Stades repères de l’abricotier. Stades repères du pêcher,” in Guide Pratique de Défense des Cultures (Paris: ACTA).

[B8] Ballester J. Socias i Company R. Arús P. Vicente M. C. (2001). Genetic mapping of a major gene delaying blooming time in almond. Plant Breed. 120, 268–270. 10.1046/j.1439-0523.2001.00604.x

[B9] Bates D. Maechler M. Bolker B. Walker S. (2015). Fitting linear mixed-effects models using lme4. J. Stat. Soft. 67 (1), 1–48. 10.18637/jss.v067.i01

[B10] Bielenberg D. G. Wang Y. Li Z. G. Zhetentyayeva T. Fan S. H. Reighard G. L. (2008). Sequencing and annotation of the evergrowing locus in peach *Prunus persica* (L.) Batsch reveals a cluster of six MAD-box transcription factors as candidate genes for regulation of terminal bud formation. Tree Genet. Genomes 4, 495–507. 10.1007/s11295-007-0126-9

[B11] Bielenberg D. G. Rauh B. Fan S. Gasic K. Abbott A. G. Reighard G. L. (2015). Genotyping by sequencing for SNP-based linkage map construction and QTL analysis of chilling requirement and bloom date in peach [*Prunus persica* (L.) Batsch]. PloS One 10 (10), e0139406. 10.1371/journal.pone.0139406 26430886 PMC4592218

[B12] Bink M. C. A. M. Boer M. P. ter Braak C. J. F. Jansen J. Voorrips R. E. van de Weg W. E. (2008). Bayesian analysis of complex traits in pedigreed plant populations. Euphytica 161, 85–96. 10.1007/s10681-007-9516-1

[B13] Bink M. C. Jansen J. Madduri M. Voorrips R. E. Durel C. E. Kouassi A. B. (2014). Bayesian QTL analyses using pedigreed families of an outcrossing species, with application to fruit firmness in apple. Theor. Appl. Genet. 127, 1073–1090. 10.1007/s00122-014-2281-3 24567047

[B14] Cachi A. M. Wünsch A. (2011). Characterization and mapping of non-S gametophytic self-compatibility in sweet cherry (*Prunus avium* L.). J. Exp. Bot. 62, 1847–1856. 10.1093/jxb/erq374 21127024

[B15] Cachi A. M. Hedhly A. Hormaza J. I. Wünsch A. (2014). Pollen tube growth in the self-compatible sweet cherry genotype, ‘Cristobalina’, is slowed down after self-pollination. Ann. Appl. Biol. 164 (1), 73–84. 10.1111/aab.12079

[B16] Cai L. Voorrips R. E. van de Weg R. Peace C. Iezzoni A. (2017). Genetic structure of a QTL hotspot on chromosome 2 in sweet cherry indicates positive selection for favorable haplotypes. Mol. Breed. 37, 85. 10.1007/s11032-017-0689-6

[B17] Cai L. Stegmeir T. Sebolt A. Zheng C. Bink M. C. A. M. Iezzoni A. (2018). Identification of bloom date QTLs and haplotype analysis in tetraploid sour cherry (*Prunus cerasus*). Tree Genet. Genomes 14, 22. 10.1007/s11295-018-1236-2

[B18] Calle A. Cai L. Iezzoni A. Wünsch A. (2018). High-density linkage maps constructed in sweet cherry (*Prunus avium* L.) using cross- and self-pollinated populations reveal chromosomal homozygosity in inbred families and non-syntenic region with the peach genome. Tree Genet. Genomes 14, 37. 10.1007/s11295-018-1252-2

[B19] Campoy J. A. Ruiz D. Allderman L. Cook N. Egea J. (2011). The fulfilment of chilling requirements and the adaptation of apricot (*Prunus armeniaca* L) in warm winter climates: An approach in Murcia (Spain) and the Western Cape (South Africa). Eur. J. Agron. 37 (1), 43–55. 10.1016/j.eja.2011.10.004

[B20] Castède S. Campoy J. A. Quero-García J. Le Dantec L. Lafargue M. Barreneche T. (2014). Genetic determinism of phenological traits highly affected by climate change in *Prunus avium*: flowering date dissection into chilling and heat requirements. New Phytol. 202, 703–715. 10.1111/nph.12658 24417538

[B21] Castède S. Campoy J. A. Le Dantec L. Quero-García J. Barreneche T. Weden B. (2015). Mapping of candidate genes involved in bud dormancy and flowering time in sweet cherry (*Prunus avium*). PloS One 10 (11), e0143250. 10.1371/journal.pone.0143250 26587668 PMC4654497

[B22] Di Guardo M. Bink M. C. A. M. Guerra W. Letschka T. Lozano L. Busatto N. (2017). Deciphering the genetic control of fruit texture in apple by multiple family-based analysis and genome-wide association. J. Exp. Bot. 68, 1451–1466. 10.1093/jxb/erx017 28338805 PMC5441909

[B23] Dirlewanger E. Quero-García J. Le Dantec L. Lambert P. Ruiz D. Dondini L. (2012). Comparison of the genetic determinism of two key phenologycal traits, flowering and maturity dates, in three *Prunus* species: peach, apricot and sweet cherry. Hered 109, 280–292. 10.1038/hdy.2012.38 PMC3477885 22828898

[B24] Fadón E. Rodrigo J. (2018). Unveiling winter dormancy through empirical experiments. Environ. Exp. Bot. 152, 28–36. 10.1016/j.envexpbot.2017.11.006

[B25] Fan S. Bielenberg D. G. Zhebentyayeva T. N. Reighard G. L. Okie W. R. Holland D. (2010). Mapping quantitative trait loci associated with chilling requirement, heat requirement and bloom date in peach (*Prunus persica*). New Phytol. 185, 917–930. 10.1111/j.1469-8137.2009.03119.x 20028471

[B26] Fresnedo-Ramírez J. Bink M. C. A. M. van de Weg E. Famula T. Crisosto C. Frett T. (2015). QTL mapping of pomological traits in peach and related species breeding germplasm. Mol. Breed. 35, 166. 10.1007/s11032-015-0357-7

[B27] Fresnedo-Ramírez J. Frett T. J. Sandefur P. J. Salgado-Rojas A. Clark J. R. Gasic K. (2016). QTL mapping and breeding value estimation through pedigree-based analysis of fruit size and weight in four diverse peach breeding programs. Tree Genet. Genomes 12, 25. 10.1007/s11295-016-0985-z

[B28] Gelman A. Rubin D. B. (1992). Inference from iterative simulation using multiple sequences. Stat. Sci. 7 (4), 472–475. 10.1214/ss/1177011136

[B29] Guan Y. Peace C. Rudell D. Verma S. Evans K. (2015). QTLs detected for individual sugars and soluble solids content in apple. Mol. Breed. 35, 135. 10.1007/s11032-015-0334-1

[B30] Hernández Mora J. R. Micheletti D. Bink M. van de Weg E. Cantín C. Nazzicari N. (2017). Integrated QTL detection for key breeding traits in multiple peach progenies. BMC Genomics 18, 404. 10.1186/s12864-017-3783-6 28583082 PMC5460339

[B31] Herrero M. Rodrigo J. Wünsch A. (2017). “Flowering, fruit set and development,” in Cherries: Botany, production and uses. Eds. Quero-García J. Iezzoni A. Lang G. (Boston, MA: CAB International), 14–35.

[B32] Herrero J. (1964). Cartografía de las variedades frutales de hueso y pepita (Aula Dei, Zaragoza: CSIC).

[B33] Howard N. P. van de Weg E. Tillman J. Tong C. B. S. Silverstein K. A. T. Luby J. J. (2018). Two QTL characterized for solf scald and soggy breakdown in apple (*Malus* × *domestica*) though pedigree-based analysis of large population of interconnected families. Tree Genet. Genomes 14, 2. 10.1007/s11295-017-1216-y

[B34] Jimenez S. Reighard G. L. Bielenberg D. G. (2010). Gene expression of DAM5 and DAM6 is suppressed by chilling temperatures and inversely correlated with bud break rate. Plant Mol. Biol. 73, 157–167. 10.1007/s11103-010-9608-5 20143130

[B35] Kass R. E. Raftery A. E. (1995). Bayes factors. J. Am. Stat. Assoc. 90, 773–795. 10.1080/01621459.1995.10476572

[B36] Kitamura Y. Habu T. Yamane H. Nishiyama S. Kajita K. Soube T. (2018). Identification of QTLs controlling chilling and heat requirements for dormancy release and bud break in Japanese apricot (*Prunus mume*). Tree Genet. Genomes 14, 33. 10.1007/s11295-018-1243-3

[B37] Lang G. A. Early J. D. Martin G. C. Darrel R. L. (1987). Endo-, para-, and endodormancy: physiological terminology and classification for dormancy research. HortScience 22, 37–377.

[B38] Leida C. Conejero A. Arbona V. Gómez-Cadenas A. Llácer G. Badenes M. L. (2012). Chilling-dependent release of seed and bud dormancy in peach associates to common changes in gene expression. PloS One 7, e35777. 10.1371/journal.pone.0035777 22590512 PMC3349671

[B39] Li Z. Reighard G. L. Abbott A. G. Bielenberg D. G. (2009). Dormancy-associated MADS genes from the EVG locus of peach [*Prunus persica* (L.) Batsch] have distinct seasonal and photoperiodic expression patterns. J. Exp. Bot. 60, 3521–3530. 10.1093/jxb/erp195 19553369 PMC2724702

[B40] Luedeling E. (2012). Climate change impacts on winter chill for temperate fruit and nut production: a review. Sci. Hortic. 144, 218–229. 10.1016/j.scienta.2012.07.011

[B41] Mahmood K. Carew J. G. Hadley P. Battey N. H. (2000). The effect of chilling and post-chilling temperature on growth and flowering of sweet cherry (*Prunus avium* L.). J. Hort. Sci. Biotechnol. 75 (5), 598–601. 10.1080/14620316.2000.11511292

[B42] Mangandi J. Verma S. Osorio L. Peres N. A. van de Weg E. Whitaker V. M. (2017). Pedigree-based analysis in multiparental population of octoploid strawberry reveals QTL alleles conferring resistance to *Phytopthora cactorum* . G3- Genes Genome Genet. 7 (6), 1707–1719. 10.1534/g3.117.042119 PMC5473751 28592652

[B43] Okie W. R. Blackburn B. (2011). Increasing chilling reduces heat requirement for floral budbreak in peach. HortScience 46, 245–252. 10.21273/HORTSCI.46.2.245

[B44] Olukolu B. A. Trainin T. Fan S. Kole C. Bielenberg D. G. Reighard G. L. (2009). Genetic linkage mapping for molecular dissection of chilling requirement and budbreak in apricot (*Prunus armeniaca* L.). Genome 52 (10), 819–828. 10.1139/g09-050 19935906

[B45] Ono K. Akagi T. Morimoto T. Wünsch A. Tao R. (2018). Genome re-sequencing of diverse sweet cherry (*Prunus avium*) individuals reveals a modifier gene mutation conferring pollen-part self-compatibility. Plant Cell Physiol. 59, 1265–1275. 10.1093/pcp/pcy068 29635538

[B46] Pauly L. Flajoulot S. Garon J. Julier B. Béguier V. Barre P. (2012). Detection of favorable alleles for plant height and crown rust tolerate in three connected populations of perennial ryegrass (*Lolium perenne* L.). Theor. Appl. Genet. 124, 1139–1153. 10.1007/s00122-011-1775-5 22234605

[B47] Piaskowski J. Hardner C. Cai L. Zhao Y. Iezzoni A. Peace C. (2018). Genomic heritability estimates in sweet cherry reveal non-additive genetic variance is relevant for industry-prioritized traits. BMC Genet. 19 (1), 23. 10.1186/s12863-018-0609-8 29636022 PMC5894190

[B48] Quilot B. Wu B. H. Kervella J. Génard M. Foulongne M. Moreau K. (2004). QTL analysis of quality traits in an advanced backcross between *Prunus persica* cultivars and the wild relative species *P. davidiana* . Theor. Appl. Genet. 109, 884–897. 10.1007/s00122-004-1703-z 15168024

[B49] R Core Team. (2017). R: a language and environment for statistical computing (Vienna: R Foundation for Statistical Computing). http://www.R-project.org/http://www.R-project.org/.

[B50] Ritter A. Iñigo S. Fernández-Calvo P. Heyndrickx K. S. Dhondt S. Shi H. (2018). The transcriptional repressor complex FRS7-FRS12 regulates flowering time and growth in *Arabidopsis* . Nat. Commun. 8, 15235. 10.1038/ncomms15235 PMC5437275 28492275

[B51] Roach J. A. Verma S. Peres N. A. Jamieson A. R. van de Weg W. E. Bink M. C. A. M. (2016). FaRXf1: a locus conferring resistance to angular leaf spot caused by *Xanthomonas fragariae* in octoploid strawberry. Theor. Appl. Genet. 129, 1191–1201. 10.1007/s00122-016-2695-1 26910360

[B52] Romeu J. F. Monforte A. J. Sanchez G. Granell A. Garcia-Brunton J. Badenes M. L. (2014). Quantitative trait loci affecting reproductive phenology in peach. BMC Plant Biol. 14, 52. 10.1186/1471-2229-14-52 24559033 PMC3941940

[B53] Rosyara U. R. Bink M. C. A. M. van de Weg E. Zhang G. Wang D. Sebolt A. (2013). Fruit size QTL identification and the prediction of parental QTL genotypes and breeding values in multiple pedigreed populations of sweet cherry. Mol. Breed. 32, 875–887. 10.1007/s11032-013-9916-y

[B54] Sánchez-Pérez R. Dicenta F. Martinez-Gomez P. (2012). Inheritance of chilling and heat requirements for flowering in almond and QTL analysis. Tree Genet. Genomes 8 (2), 379–389. 10.1007/s11295-011-0448-5

[B55] Salazar J. A. Ruiz D. Egea J. Martínez-Gómez P. (2013). Transmission of fruit quality traits in apricot (*Prunus armeniaca* L.) and analysis of linked quantitative trait loci (QTLs) using simple sequence repeat (SSR) markers. Plant Mol. Biol. Rep. 31 (6), 1506–1517. 10.1007/s11105-013-0625-9

[B56] Salazar J. A. Ruiz D. Campoy J. A. Sánchez-Pérez R. Crisosto C. H. Martínez-García P. J. (2014). Quantitative Trait Loci (QTL) and Mendelian Trait Loci (MTL) analysis in *Prunus*: a breeding perspective and beyond. Plant Mol. Biol. Rep. 32, 1–18. 10.1007/s11105-013-0643-7

[B57] Salazar J. A. Ruiz D. Campoy J. A. Tartarini S. Dondini L. Martínez-Gómez P. (2016). Inheritance of reproductive phenology traits and relates QTL identification in apricot. Tree Genet. Genomes 12, 71. 10.1007/s11295-016-1027-6

[B58] Sorensen D. Gianola D. (2002). Likelihood, Bayesian, and MCMC methods in quantitative genetics (New York: Springer-Verlag).

[B59] Tabuenca M. C. (1983). Winter chilling requirements of cherry varieties (Comunicaciones SECH: I Congreso Nacional de la Sociedad Española de Ciencias Hortícolas (Valencia)), 661–667.

[B60] Trainin T. Bar-Ya’akov I. Holland D. (2013). ParSOC1, a MADS-box gene closely related to Arabidopsis AGL20/SOC1, is expressed in apricot leaves in a diurnal manner and is linked with chilling requirements for dormancy break. Tree Genet. Genomes 9 (3), 753–766. 10.1007/s11295-012-0590-8

[B61] Verde I. Jenkins J. Dondini L. Micali S. Pagliarani G. Vendramin E. (2017). The Peach v2.0 release: high-resolution linkage mapping and deep resequencing improve chromosome-scale assembly and contiguity. BMC Genomics 18, 1–18. 10.1186/s12864-017-3606-9 28284188 PMC5346207

[B62] Voorrips R. E. (2007). Pedimap: Software for visualization of genetic and phenotypic data in pedigrees (Wagenigen, the Netherlands: Plant Research International). 10.1093/jhered/ess060 PMC3510005 23087384

[B63] Wünsch A. Hormaza J. I. (2004). Genetic and molecular analysis in Cristobalina sweet cherry, a spontaneous self-compatible mutant. Sex Plant Reprod. 17, 203–210. 10.1007/s00497-004-0234-8

[B64] Wang D. Karle R. Iezzoni A. F. (2000). QTL analysis of flower and fruit traits in sour cherry. Theor. Appl. Genet. 100 (3–4), 535–544. 10.1007/s001229900121

[B65] Wang D. Gao Z. Du P. Xiao W. Tan Q. Chen X. (2016). Expression of ABA metabolism-related genes suggests similarities and differences between seed dormancy and bud dormancy of peach (*Prunus persica*). Front. Plant Sci. 6, 1248. 10.3389/fpls.2015.01248 26793222 PMC4707674

[B66] Yamane H. Ooka T. Jotatsu H. Hosaka Y. Sasaki R. Tao R. (2011). Expressional regulation of PpDAM5 and PpDAM6 peach (*Prunus persica*) dormancy-associated MADS-box genes, by low temperature and dormancy-breaking reagent treatment. J. Exp. Bot. 62, 3481–3488. 10.1093/jxb/err028 21378115 PMC3130173

[B67] Zhebentyayeva T. N. Fan S. Chandra A. Bielenberg D. G. Reighard G. L. Okie W. R. (2014). Dissection of chilling requirement and bloom date QTLs in peach using a whole genome sequencing of sibling trees from an F2 mapping population. Tree Genet. Genomes 10, 35–51. 10.1007/s11295-013-0660-6

