# Supplementary material for: Genetic Dissection of Bloom Time in Low Chilling Sweet Cherry (Prunus avium L.) Using a Multi-Family QTL Approach
Source: Front Plant Sci. 2020 Jan 9;10:1647. doi: 10.3389/fpls.2019.01647 (PMC6962179; doi:10.3389/fpls.2019.01647)
Supplement: Supplementary file 1 [file DataSheet_1.docx]

Supplementary Material

**Supplementary Table S1** a) Bloom time (BT) of parental cultivars in Julian days (JD) per year. b) BT mean, standard deviation (SD), minimum (Min) and maximum (Max) values, and number of individuals phenotyped each year (N), per year and family. Broad-sense heritability (*H^2^*) of each family for the 4 years.

**a)**

|  | **‘Ambrunés’** | **‘BC-8’** | **‘Brooks’** | **‘Cristobalina’** | **‘Lambert’** | **‘Vic’** |
| --- | --- | --- | --- | --- | --- | --- |
| **2015** | 92 | 96 | 92 | 74 | 101 | 97 |
| **2016** | 92 | - | 92 | 58 | 103 | 99 |
| **2017** | 76 | - | 78 | 64 | 85 | 82 |
| **2018** | 94 | - | 98 | 78 | 101 | 101 |

**b)**

|  |  | **A×C** | **B×C** | **B×C F2** | **C×C** | **L×C** | **V×C** |
| --- | --- | --- | --- | --- | --- | --- | --- |
| **2015** | **Mean ± SD** | 92.2±3.7 | 93.6±3.9 | 88.4±4.5 | 78.0±4.4 | 94.0±3.2 | 92.0±3.5 |
|  | **Min** | 87 | 84 | 80 | 71 | 88 | 85 |
|  | **Max** | 99 | 100 | 96 | 87 | 97 | 102 |
|  | **N** | 36 | 24 | 28 | 81 | 13 | 76 |
| **2016** | **Mean ± SD** | 89.1±3.2 | 88.9±3.3 | 90.8±4.5 | 71.2±8.7 | 93.4±3.6 | 94.4±4.0 |
|  | **Min** | 82 | 83 | 83 | 60 | 88 | 87 |
|  | **Max** | 95 | 96 | 98 | 84 | 100 | 105 |
|  | **N** | 30 | 21 | 28 | 84 | 14 | 76 |
| **2017** | **Mean ± SD** | 76.4±2.4 | 74.5±2.6 | 75.5±4.2 | 67.4±2.7 | 79.1±3.1 | 78.5±2.4 |
|  | **Min** | 71 | 70 | 68 | 62 | 75 | 73 |
|  | **Max** | 82 | 80 | 83 | 74 | 85 | 87 |
|  | **N** | 39 | 24 | 45 | 90 | 14 | 141 |
| **2018** | **Mean ± SD** | 92.4±3.5 | 91.6±2.3 | 94.4±4.7 | 83.6±4.6 | 94.9±3.2 | 95.5±2.5 |
|  | **Min** | 87 | 87 | 86 | 73 | 89 | 89 |
|  | **Max** | 99 | 94 | 104 | 93 | 100 | 104 |
|  | **N** | 39 | 21 | 50 | 91 | 14 | 152 |
|  | ***H^2^*** | 0.90 | 0.85 | 0.96 | 0.95 | 0.96 | 0.91 |

**Supplementary Table S2** Pearson correlation coefficients of BT among years (2015 to 2018) and BLUP values.

|  | **BT 15** | **BT 16** | **BT 17** | **BT 18** | **BT BLUP** |
| --- | --- | --- | --- | --- | --- |
| **BT 15** | 1 | 0.934^*^ | 0.904^*^ | 0.897^*^ | 0.962^*^ |
| **BT 16** |  | 1 | 0.916^*^ | 0.948^*^ | 0.984^*^ |
| **BT 17** |  |  | 1 | 0.938^*^ | 0.955^*^ |
| **BT 18** |  |  |  | 1 | 0.966^*^ |
| **BT BLUP** |  |  |  |  | 1 |

* indicates significant correlation at *P<*0.01.

**Supplementary Table S3** Number of polymorphic SNPs mapped per progeny and used for QTL analysis.

|  | **A×C** | **B×C** | **B×C F2** | **C×C** | **L×C** | **V×C** | **Total** |
| --- | --- | --- | --- | --- | --- | --- | --- |
| **Chr 1** | 231 | 216 | 135 | 85 | 211 | 257 | **307** |
| **Chr 2** | 131 | 126 | 71 | 99 | 141 | 140 | **156** |
| **Chr 3** | 77 | 97 | 57 | 56 | 85 | 79 | **130** |
| **Chr 4** | 95 | 107 | 55 | 72 | 95 | 98 | **137** |
| **Chr 5** | 66 | 72 | 47 | 50 | 53 | 93 | **128** |
| **Chr 6** | 86 | 97 | 61 | 59 | 102 | 110 | **137** |
| **Chr 7** | 69 | 43 | 48 | 7 | 50 | 62 | **131** |
| **Chr 8** | 88 | 75 | 50 | 44 | 86 | 74 | **143** |
| **WG^*^** | 843 | 833 | 524 | 472 | 823 | 913 | **1269** |

^*^WG: Whole Genome

**Supplementary Table S4** Genetic map used for QTL analysis.

**Supplementary Table S5** Haplotypes of QTLs *qP-BT1.1^m^* and *qP-BT2.1^m^* in parental cultivars and ancestors.

|  |  | **Haplotypes** | | | | | | | | | | | | | | | | | | | | | | |
| --- | --- | --- | --- | --- | --- | --- | --- | --- | --- | --- | --- | --- | --- | --- | --- | --- | --- | --- | --- | --- | --- | --- | --- | --- |
| **QTL** | **SNP** | **'Ambrunés'** | | **'BC-8'** | | **'Bing'** | | **'Brooks'** | | **'Burlat'** | | **'Cristobalina'** | | **'Lambert'** | | **'Napoleon'** | | **'Rainier'** | | **'Van'** | | **'Vic'** | |  |
|  |  | ***H1-a*** | ***H1-b*** | ***H1-a*** | ***H1-c*** | ***H1-a*** | ***H1-a*** | ***H1-a*** | ***H1-a*** | ***H1-b*** | ***H1-a*** | ***H1-c*** | ***H1-c*** | ***H1-b*** | ***H1-a*** | ***H1-a*** | ***H1-b*** | ***H1-a*** | ***H1-a*** | ***H1-a*** | ***H1-b*** | ***H1-a*** | ***H1-b*** |  |
| ***qP-BT1.1^m^*** | ss490548667 | A | B | A | B | A | A | A | A | B | A | B | B | B | A | A | B | A | A | A | B | A | B |  |
|  | ss490546979 | B | B | B | A | B | B | B | B | B | B | A | A | B | B | B | B | B | B | B | B | B | B |  |
|  | ss490548655 | B | A | B | B | B | B | B | B | A | B | B | B | A | B | B | A | B | B | B | A | B | A |  |
|  | ss490548643 | B | B | B | A | B | B | B | B | B | B | A | A | B | B | B | B | B | B | B | B | B | B |  |
|  |  |  |  |  |  |  |  |  |  |  |  |  |  |  |  |  |  |  |  |  |  |  |  |  |
|  |  |  |  |  |  |  |  |  |  |  |  |  |  |  |  |  |  |  |  |  |  |  |  |  |
|  |  |  |  |  |  |  |  |  |  |  |  |  |  |  |  |  |  |  |  |  |  |  |  |  |
|  |  | **Haplotypes** | | | | | | | | | | | | | | | | | | | | | | |
| **QTL** | **SNP** | **'Ambrunés'** | | **'BC-8'** | | **'Bing'** | | **'Brooks'** | | **'Burlat'** | | **'Cristobalina'** | | **'Lambert'** | | **'Napoleon'** | | **'Rainier'** | | **'Van'** | | **'Vic'** | |  |
|  |  | ***H2-g*** | ***H2-a*** | ***H2-c*** | ***H2-e*** | ***H2-b*** | ***H2-d*** | ***H2-d*** | ***H2-c*** | ***H2-b*** | ***H2-c*** | ***H2-e*** | ***H2-f*** | ***H2-i*** | ***H2-d*** | ***H2-d*** | ***H2-k*** | ***H2-a*** | ***H2-d*** | ***H2-a*** | ***H2-d*** | ***H2-b*** | ***H2-h*** |  |
| ***qP-BT2.1^m^*** | ss490559076 | B | B | B | B | B | A | A | B | B | B | B | B | B | A | A | B | B | A | B | A | B | B |  |
|  | ss490550443 | A | A | A | A | A | B | B | A | A | A | A | B | A | B | B | A | A | B | A | B | A | B |  |
|  | ss490550465 | A | A | A | A | B | A | A | A | B | A | A | A | A | A | A | B | A | A | A | A | B | A |  |
|  | ss490550493 | B | A | B | B | A | B | B | B | A | B | B | B | B | B | B | A | A | B | A | B | A | B |  |
|  | ss490550497 | B | B | B | B | B | A | A | B | B | B | B | B | B | A | A | B | B | A | B | A | B | B |  |
|  | ss490550501 | B | A | B | B | A | B | B | B | A | B | B | B | B | B | B | A | A | B | A | B | A | B |  |
|  | ss490550517 | B | B | B | A | B | B | B | B | B | B | A | B | B | B | B | B | B | B | B | B | B | B |  |
|  | ss490550521 | A | B | A | A | B | A | A | A | B | A | A | A | B | A | A | B | B | A | B | A | B | B |  |
|  | ss490550529 | A | B | B | A | B | B | B | B | B | B | A | B | A | B | B | B | B | B | B | B | B | A |  |
|  | ss490550577 | A | B | A | A | B | B | B | A | B | A | A | A | B | B | B | B | B | B | B | B | B | B |  |
|  | ss490550588 | B | B | B | B | B | A | A | B | B | B | B | B | B | A | A | A | B | A | B | A | B | B |  |
|  | ss490550626 | B | A | B | B | B | A | A | B | B | B | B | B | B | A | A | A | A | A | A | A | B | B |  |
|  | ss490550731 | B | B | B | B | B | A | A | B | B | B | B | B | B | A | A | A | B | A | B | A | B | B |  |
|  | ss490547648 | B | A | B | B | A | A | A | B | A | B | B | B | A | A | A | A | A | A | A | A | A | A |  |

**
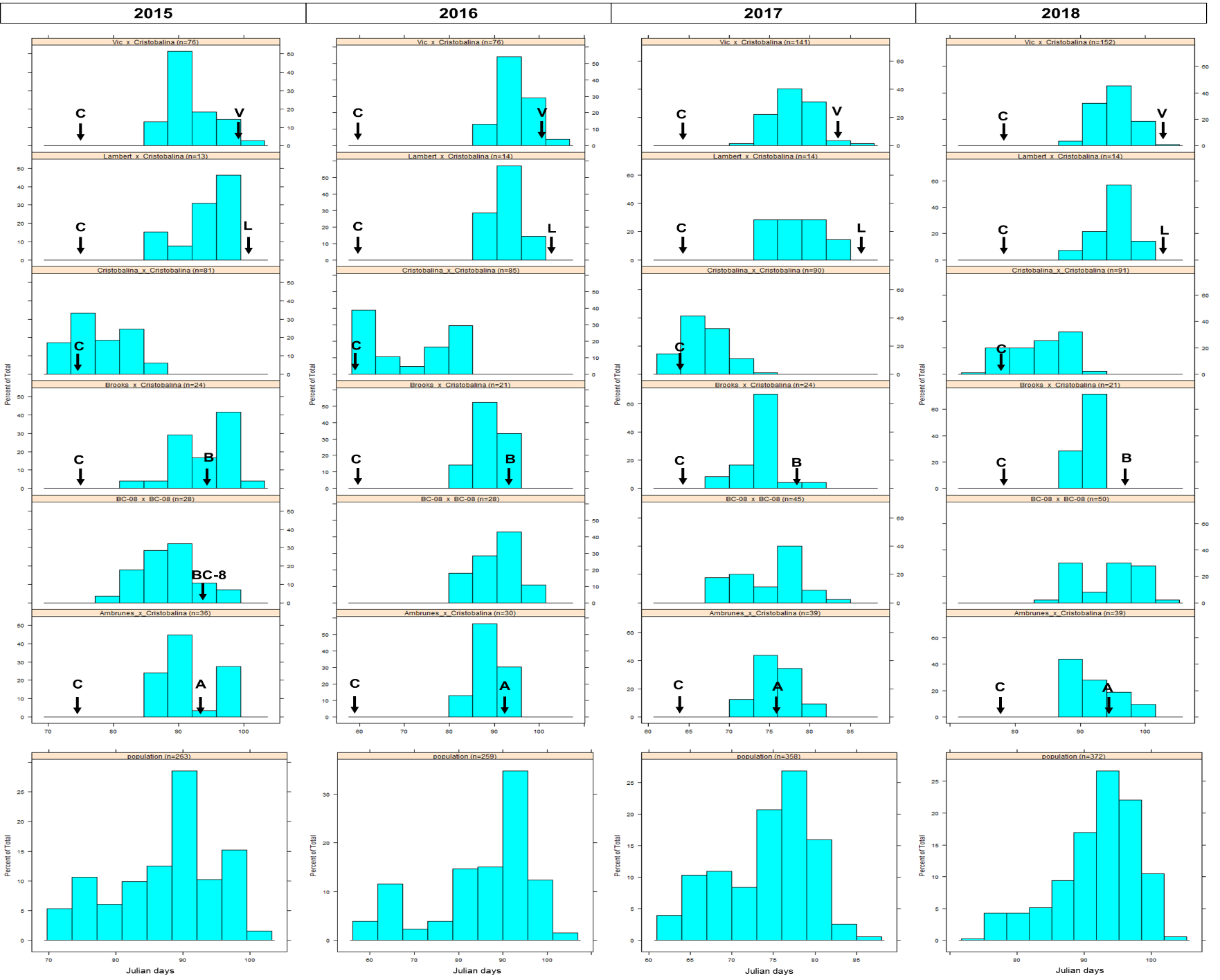
**

**Supplementary Figure S1**

Frequency distribution of single years (2015-2018) bloom time values of six population and all plant material analyzed. Letter with arrows indicate parents’ bloom time; ‘Ambrunés’ (A), ‘Brooks’ (B), ‘B×C-08’ (BC-8), ‘Cristobalina’ (C), ‘Lambert’ (L) and ‘Vic’ (V).

**
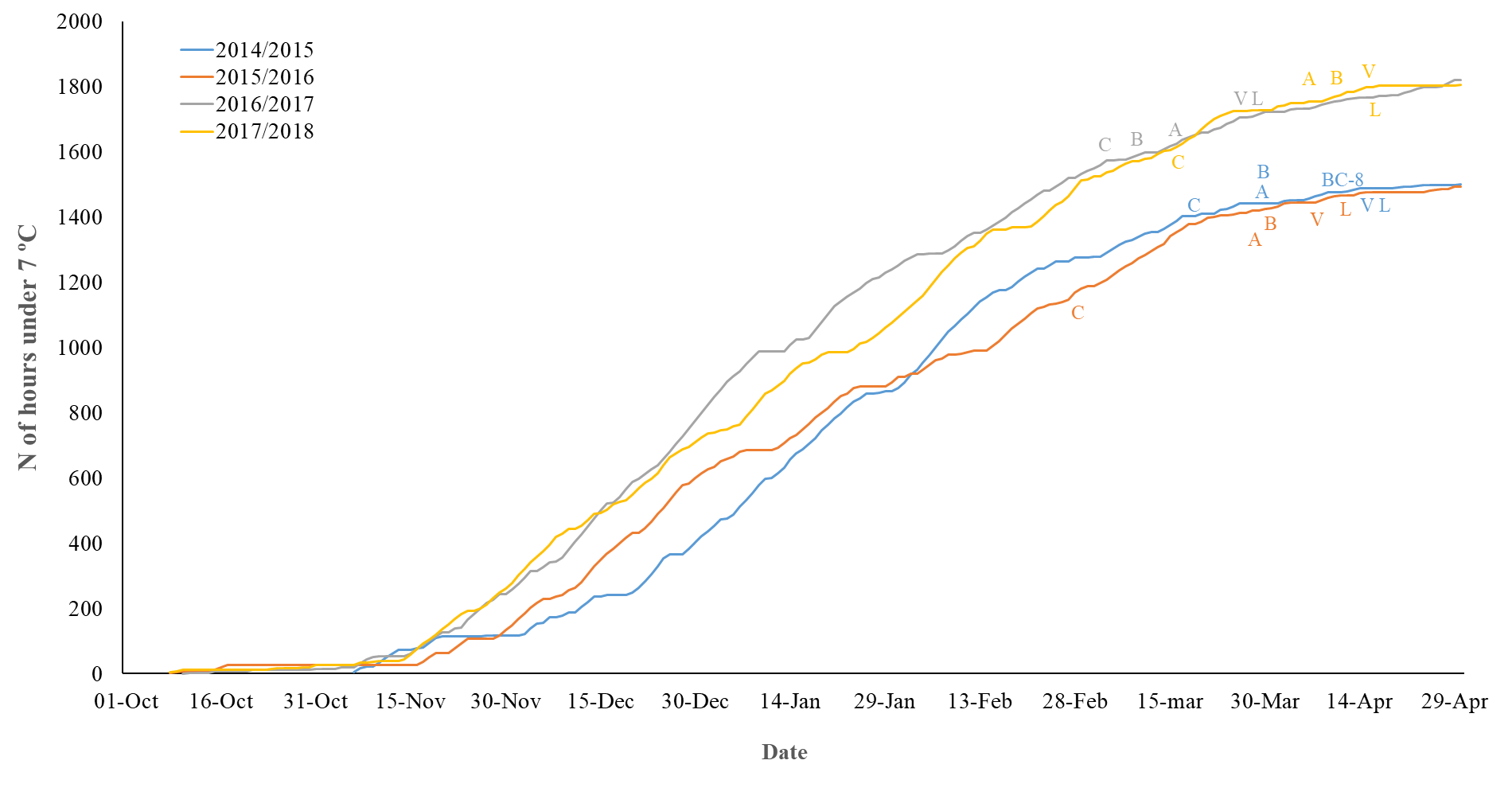
**

**Supplementary Figure S2**

Accumulated winter chilling (hours below 7 ºC^1^), and bloom dates of parental cultivars during the four years of analysis. ‘Ambrunés’ (A), ‘Brooks’ (B), ‘Cristobalina’ (C), ‘Lambert’ (L), ‘Vic’ (V) and ‘BC-08’ (BC-8). ^1^Data from: ‘Estación 34-Montañana’, ‘Datos Meteorológicos’, ‘Oficina del Regante’, ‘Sociedad Aragonesa de Gestión Agroambiental (Sarga)’, ‘Gobierno de Aragón’. [http://aplicaciones.aragon.es/oresa/datosMeteorologicos.inicio.do?sm= 2060](http://aplicaciones.aragon.es/oresa/datosMeteorologicos.inicio.do?sm=%202060)

**
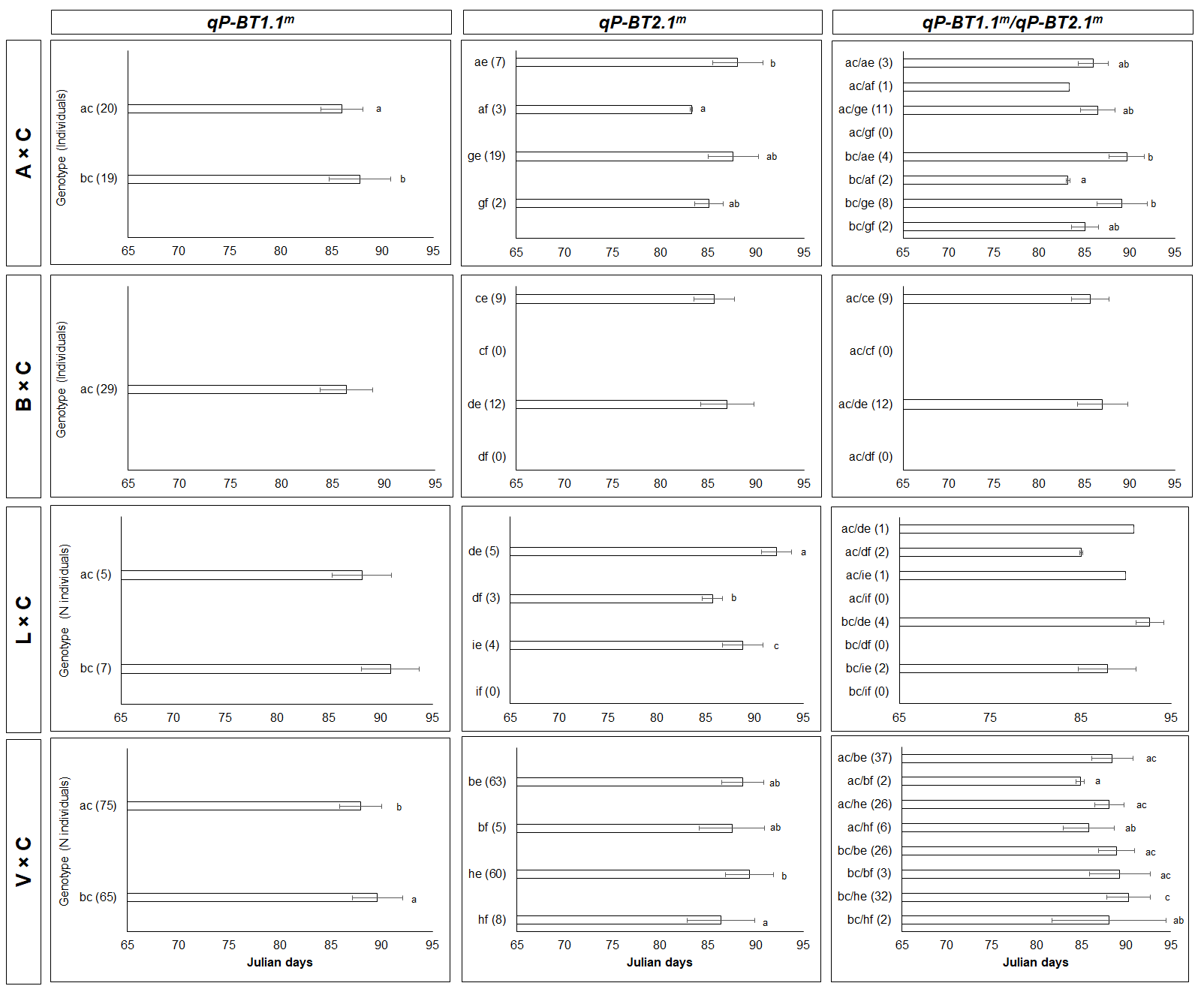
**

**Supplementary Figure S3**

BT (in JDs) mean genotype values of major QTLs detected and their interaction (*qP-BT1.1^m^*, *qP-BT2.1^m^* and both) in segregating classes of F_1_ populations analysed. Significant differences between genotypes are indicated by different letters (P<0.05).
